# Intramolecular ring-opening from a CO_2_-derived nucleophile as the origin of selectivity for 5-substituted oxazolidinone from the (salen)Cr-catalyzed [aziridine + CO_2_] coupling†
†Electronic supplementary information (ESI) available: Detailed descriptions of the computational investigations; experimental procedures for the catalytic reactions; characterization data for the oxazolidinone products; computational evaluations of selected alternative mechanisms; coordinates and vibrational frequencies of investigated structures. See DOI: 10.1039/c4sc02785j


**DOI:** 10.1039/c4sc02785j

**Published:** 2014-11-21

**Authors:** Debashis Adhikari, Aaron W. Miller, Mu-Hyun Baik, SonBinh T. Nguyen

**Affiliations:** a Department of Chemistry and the International Institute for Nanotechnology , Northwestern University , 2145 Sheridan Road , Evanston , IL 60208-3113 , USA . Email: stn@northwestern.edu; b Department of Chemistry , Indiana University , 800 East Kirkwood Avenue , Bloomington , IN 47405 , USA . Email: mbaik@indiana.edu; c Department of Materials Chemistry , Korea University , Jochiwon-eup , Sejong-si , 339-700 , South Korea

## Abstract

The (salen)Cr-catalyzed [aziridine + CO_2_] coupling to form oxazolidinone was found to exhibit excellent selectivity for the 5-substituted oxazolidinone product in the absence of any cocatalyst. Quantum mechanical calculations suggest that the preferential opening of the substituted C–N bond of the aziridine over the unsubstituted C–N bond is a key factor for this selectivity, a result that is supported by experiment with several phenyl-substituted aziridines. In the presence of external nucleophile such as dimethyl aminopyridine (DMAP), the reaction changes pathway and the ring-opening process is regulated by the steric demand of the nucleophile.

## Abstract 2

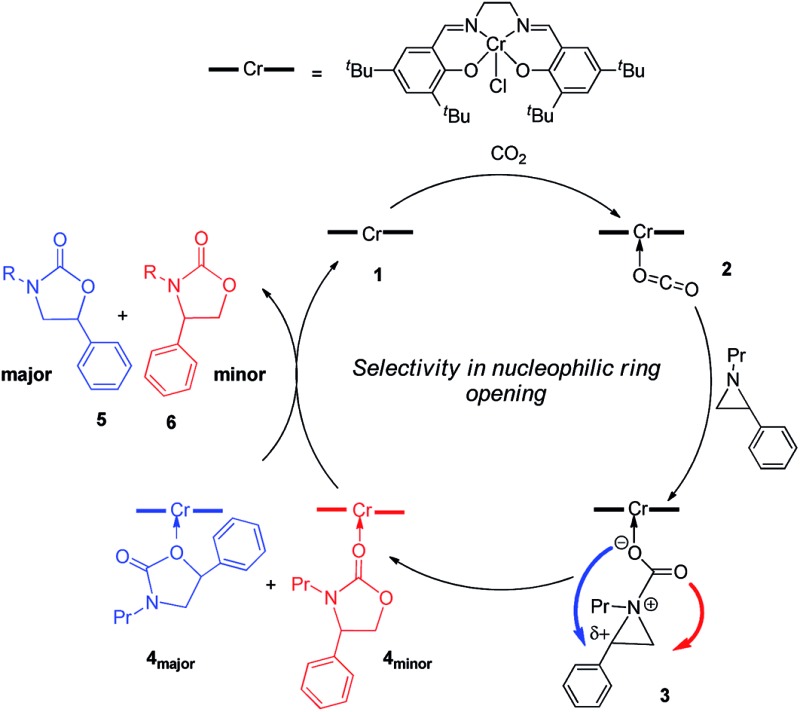
The (salen)Cr-catalyzed [aziridine + CO_2_] coupling to form oxazolidinone was found to exhibit excellent selectivity for the 5-substituted oxazolidinone product in the absence of any cocatalyst.

## Introduction

Oxazolidinones constitute an important class of organic molecules with significant biological relevance,^1^ for example as antibiotic agents against various Gram-positive bacteria,^2^ and rich reactive chemistry that can be exploited in the syntheses of challenging natural products, pharmaceutical agents, and chiral ligands.^3^–^5^ An important subgroup of this class, the 1,3-oxazolidin-2-ones that are also known as Evans chiral auxiliaries, has been utilized widely to promote various organic reactions, such as alkylation,^6^,^7^ aldol condensation,^8^,^9^ Diels–Alder reaction,^3^,^4^ *etc.* Traditional oxazolidinone syntheses often rely on the use of phosgene and reactive derivatives of carbonic acid, which is not atom-economical and can limit the scope of their utility.^10^–^12^ In this respect, the catalytic coupling of aziridines and CO_2_ is an attractive alternative that can exploit the easy accessibility of a broad range of substituted aziridines and CO_2_.^13^,^14^ Surprisingly, little effort has been focused on this reaction in comparison to the tremendous attention that has been paid to the analogous coupling of epoxide and CO_2_.^15^–^24^

During the past decade, a handful of catalysts—including DMAP,^25^ alkali metal halide,^26^,^27^ tetraalkylammonium halide,^26^,^28^ and iodine^29^—have been utilized to couple aziridines and CO_2_ into 4-substituted oxazolidinone or an unselective mixture of 5- and 4-substituted oxazolidinones. We also reported the use of [(salen)Cr^III^Cl + DMAP] catalyst in the facile conversion of a range of aziridines into 5-substituted and 4-substituted oxazolidinones with selectivity up to 20 : 1 favoring the 5-substituted isomer (eqn (1)).^30^ While there are several reports of selective oxazolidinone formation from aziridine and CO_2_, the observed selectivity was only moderately in favor of 4-substituted oxazolidinone, consistent with the opening of the aziridine ring at the less substituted position.^31^,^32^ From this perspective, the high selectivity favoring 5-substituted oxazolidinone for the [(salen)Cr^III^Cl + DMAP] catalyst system is quite unique and we proposed that this is a consequence of a Lewis-acid activation that favored ring-opening at the carbon stabilized by the aryl substituent.^30^
1

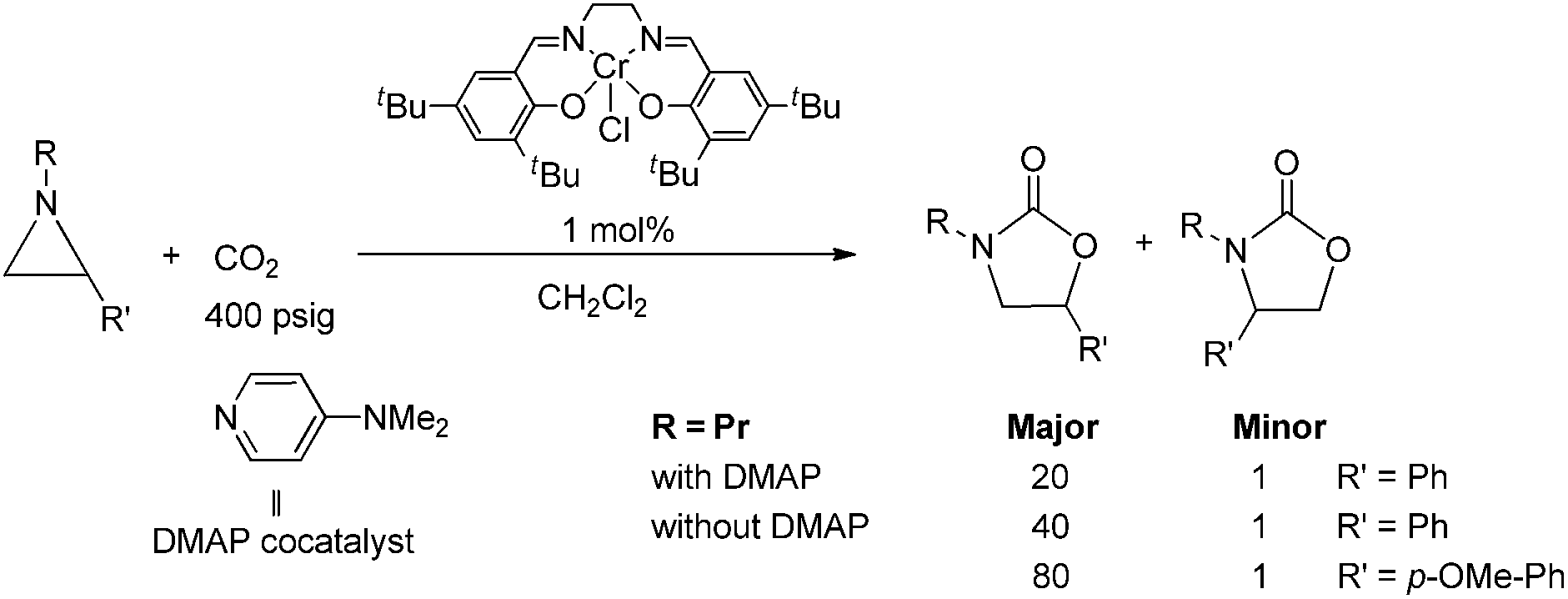




**Fig. 1:**
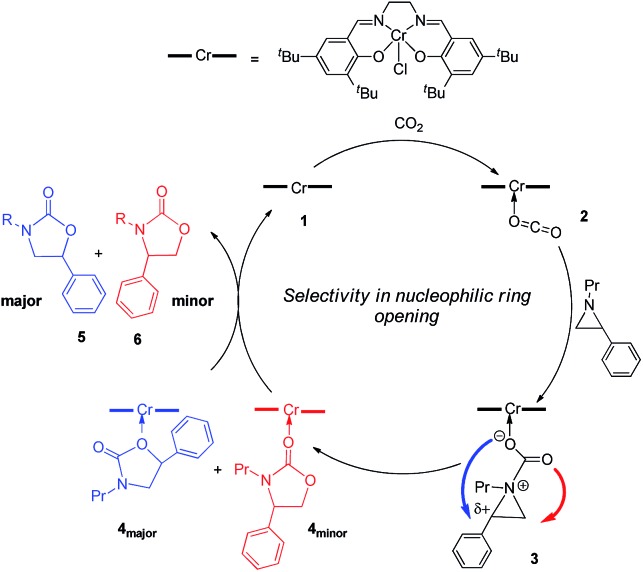
A proposed catalytic cycle for the formation of 5- and 4-substituted oxazolidinones from the coupling of aziridine and CO_2_ in the sole presence of the (salen)Cr^III^Cl catalyst. The pathway indicated by the blue arrow produces the major product.

Interestingly, (salen)Cr^III^Cl was even more selective for reaction 1 in the absence of DMAP cocatalyst: the conversion of *N*-propyl-2-phenylaziridine to the corresponding 5-substituted oxazolidinone proceeds with a selectivity of 40 : 1, albeit with a slightly slower rate than that for the DMAP-cocatalyzed reaction.^30^ This selectivity is increased to 80 : 1 when the substrate is the electron-rich *N*-propyl-2-(*p*-methoxyphenyl)aziridine (see below). These data are in stark contrast to the analogous [epoxide + CO_2_] coupling^33^,^34^ where the Lewis-basic DMAP cocatalyst is crucial for the successful completion of the reaction. Intrigued by this observation, we set out to investigate the mechanism of reaction 1 using a comprehensive array of theoretical calculations to corroborate with experimental results and pinpoint the key parameters that dictate the observed selectivity and reactivity. Herein, we propose a novel mechanism for reaction 1 that features an initial binding of CO_2_ to the (salen)Cr^III^ center (Fig. 1). This activation allows for the aziridine substrate to attack the CO_2_ carbon to form a (salen)Cr^III^(aziridiniumcarbamate) intermediate (**3**). The CO_2_-derived oxygen nucleophile of the carbamate moiety can then intramolecularly ring-open the tethered aziridine substrate. The uniqueness of this mechanism lies in the key presence of the CO_2_-coordinated intermediate **2** and the ability of the carbamate oxygen nucleophile to regulate the oxazolidinone selectivity by preferentially opening one of the two available C–N bonds in an *intramolecular* fashion, depending on the ability of the substituents at the aziridine C^2^ to stabilize the developing cationic charges.

## Results and discussion

### Proposed mechanism for the (salen)Cr^III^-catalyzed coupling of CO_2_ and aziridine in the absence of the DMAP cocatalyst

Our quantum mechanical calculations (DFT, M06 level of theory^35^,^36^) reveal that under the high-pressure conditions employed in the closed experimental system shown in eqn (1), dissolved CO_2_ can weakly bind to the highly Lewis-acidic (salen)Cr^III^Cl center and cause a slight polarization of the electron density in the coordinated C=O bond. This results in an increase in the electrophilicity of the CO_2_ carbon and renders it susceptible to a nucleophilic attack by the phenyl aziridine substrate to form intermediate **3** (Fig. 1). The desired oxazolidinone product is then formed through a combination of synchronous, concerted three-membered aziridine ring-opening and five-membered ring-closing processes. Key to the observed high selectivity for the 5-substituted oxazolidinone **5** is an increase in the carbocationic character of the carbon bearing the phenyl substituent, leading to an N–C^2^ bond cleavage on the phenyl-substituted side of the aziridine ring and resulting in the major product after ring closure. The alternative N–C^3^ bond cleavage at the unsubstituted carbon is kinetically unfavorable and affords the minor product **6** in a very small amount.

As mentioned above, the binding of CO_2_ to the Lewis-acidic (salen)Cr^III^Cl center results in a polarization of the coordinated C=O bond, which slightly elongates (1.17 Å) over the other C=O bond (1.16 Å).^37^ This electronic perturbation causes a slight increase in the electrophilicity of the CO_2_ carbon (its electrostatic-potential (ESP)-fitted charge increases to 0.73 from 0.69), rendering it easier to undergo attack by the phenyl aziridine substrate. Our quantum mechanical calculations suggest that this event is favored enthalpically by 7.5 kcal mol^–1^, but is canceled out by the translational entropic penalty to afford a solvation-corrected Gibbs free energy of –0.1 kcal mol^–1^, measured from the initial lowest-energy reference state of the system (catalyst and substrates being at infinite distance).

While the activation of CO_2_ by (salen)Cr^III^Cl, as shown in Fig. 1, is favored by the high pressure of CO_2_ employed in our experiments, it can be inhibited by the direct binding of the Lewis-basic aziridine substrate to the Cr center. Such coordination can competitively retard the rate of the catalytic cycle, especially in the absence of a cocatalyst that can ring-open the coordinated substrate. Indeed, our calculations reveal that while the Lewis acid–Lewis base interaction between (salen)Cr^III^Cl and the aziridine substrate does significantly activate the aziridine ring (see ESI, section S4†), it is not strong enough to induce its spontaneous opening. We note in passing that in the presence of DMAP, a similar Lewis acid–Lewis base competitive binding can also occur between the Cr center and DMAP. However, in this case DMAP can also serve as the cocatalyst to ring-open the coordinated substrate and lead to the products through another pathway (see “The Effect of DMAP on isomer selectivity” below).

**Fig. 2:**
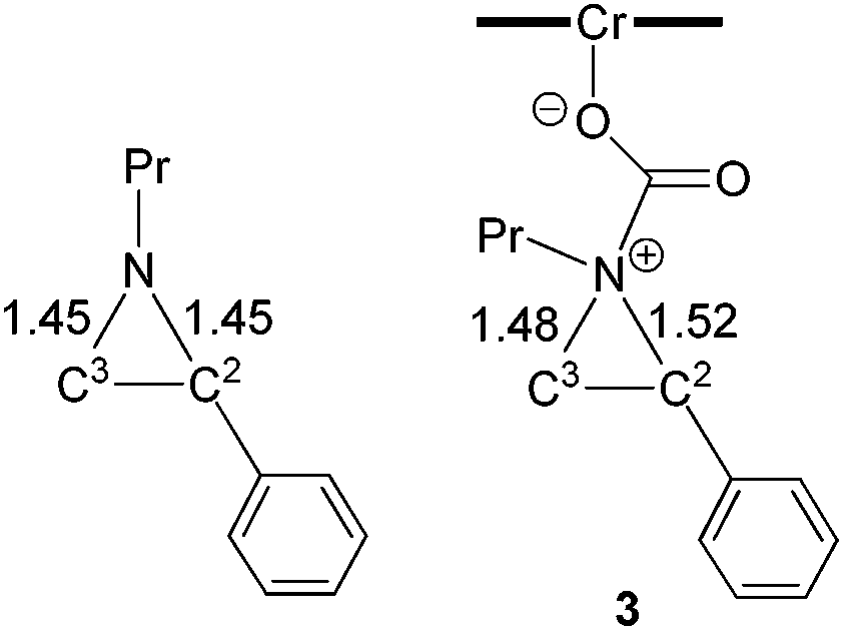
The computed structures of *N*-propyl aziridine (left) and intermediate **3** (right) after aziridine attack on the electrophilic carbon of the [(salen)Cr^III^Cl] ← O=C=O intermediate. The bond lengths are in Å.

The nucleophilic attack of the aziridine substrate on the activated CO_2_ carbon generates a (salen)Cr^III^Cl-coordinated alkoxide intermediate **3** (Fig. 2), where CO_2_ is effectively complexed between the metal center and the aziridine. A transition state (**2-TS**) for this process can be located at an energy of 14.6 kcal mol^–1^. Consistent with such a nucleophilic attack, the linear CO_2_ becomes significantly bent to 157.8°, with noticeable elongations of both “C=O” bonds (∼0.03 Å). The formation of intermediate **3** is energetically uphill by 8.7 kcal mol^–1^ from the initial lowest-energy state. (As expected, such a process is highly dependent on the nucleophilicity of the nitrogen lone pair: *N*-tosyl-2-methylaziridine, whose nitrogen lone pair is strongly delocalized into the tosyl group, is unreactive under our experimental coupling condition.) At this intermediate stage, the coordinated aziridine retains its three-membered ring structure despite significant elongations of both substituted and unsubstituted C–N bonds (Fig. 2). Notably, the phenyl-substituted N–C^2^ bond in **3** is substantially more elongated (to 1.52 Å from 1.45 Å) compared to the unsubstituted N–C^3^ bond (to 1.48 Å from 1.45 Å). This differential elongation is larger than the bond lengths change when aziridine binds directly to the Cr^III^ center (see ESI, section S4†) and can be considered as the first step to activate the aziridine ring, allowing for subsequent electronic polarization and charge development to occur. The result is a preferential ring-opening on the more elongated N–C^2^ bond. We note that our proposed mode for CO_2_ complexation, between the (salen)Cr^III^Cl center and the aziridine substrate, does not require the opening of a new coordination site from distorting the salen ligand, as proposed by Luinstra and coworkers for the coupling of CO_2_ and epoxide.^38^ In our hands, the free-energy calculations for such a ligand framework distortion process only resulted in sizable energy penalties.

### Selectivity prediction for the (salen)Cr^III^-catalyzed coupling of CO_2_ and aziridine in the absence of the DMAP cocatalyst

**Fig. 3:**
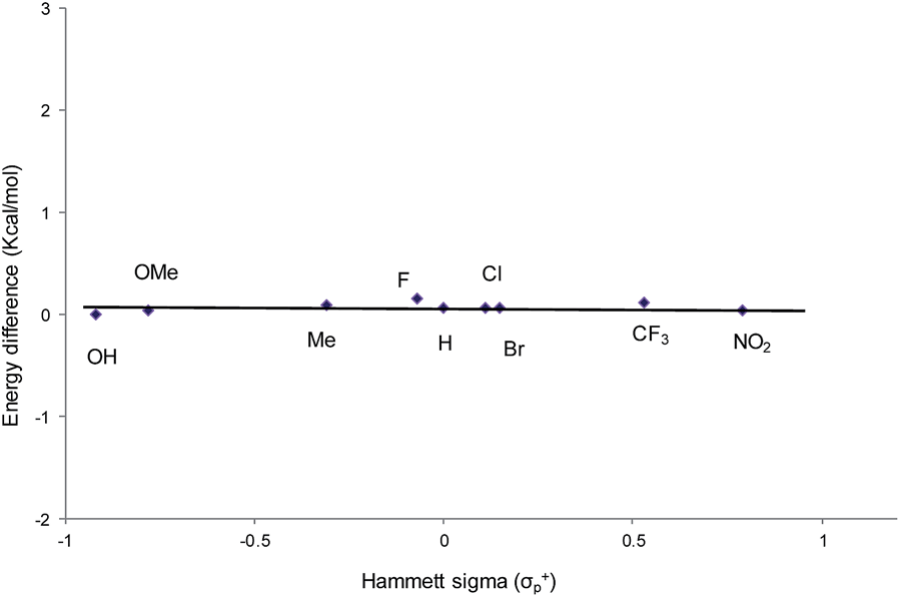
A comparison of the difference in ground-state energies between *N*-propyl-5-aryl- and *N*-propyl-4-aryl oxazolidinones with different *p*-substituents. Conformational geometries were optimized using DFT and the M06/cc-pVTZ(-f)//M06/LACVP** parameterization scheme.

From a transition-state consideration, the aforementioned elongation of the N–C^2^ bond in the aziridine-attacked intermediate **3** should logically lead to a selective formation of the 5-oxazolidinone product. To verify whether the observed selectivity of reaction 1 has a thermodynamic component, we evaluated the difference in ground-state energies of nine pairs of 5- and 4-aryl-*N*-propyl oxazolidinones comprising a broad range of para (*p*)-substituted phenyl groups. The apparent insensitivity of this difference to electronic changes in the aryl substituent (Fig. 3) suggests that the selectivity of reaction 1 is not a product-based ground-state effect.

**Fig. 4:**
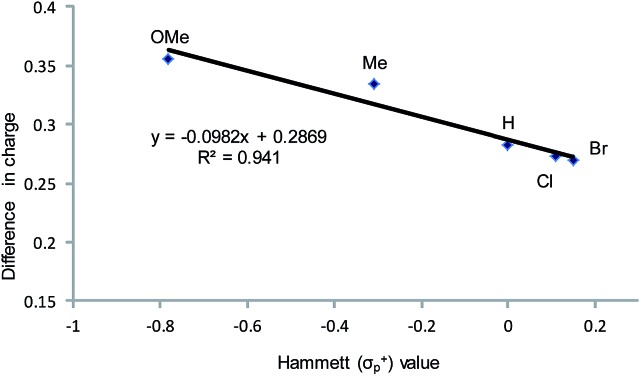
A plot of the differences in charge between C^2^ and C^3^ of the coordinated aziridine in intermediate **3** against the Hammett *σ*_p_^+^ values^39^ showing a strong correlation (*R*^2^ = 0.94 for the best fit line), suggesting a significant influence of the *p*-substituent on the developing carbocationic charges on C^2^. This trend agrees with the experimentally observed selectivity for reaction 1 in the absence of DMAP cocatalyst (see Fig. 9 below), where electron-donating substituents exhibit higher selectivity.

As indicated in Fig. 1, intermediate **3** contains two different CO_2_-derived nucleophilic sites, namely the alkoxide and carbonyl oxygens, either of which can ring-open the aziridine to form the 5- and 4-substituted oxazolidinones (*via* attacking at the substituted and unsubstituted carbon centers, respectively). Since this process is completely intramolecular, the carbon with higher partial positive character is more likely to undergo nucleophilic attack. Thus, the presence of electron-donating (or -withdrawing) *p*-substituents on the aziridine phenyl rings can be expected to greatly influence this process *via* stabilization (or destabilization) of the developing carbocationic charge at the C^2^ center. To verify this, we evaluated the differences in charges between C^2^ and C^3^ centers for five different analogs of **3** where the phenyl groups of the coordinated aziridine rings possess *p*-substituents ranging from electron-donating to -withdrawing (Table 1). When these charge differences are plotted against the corresponding Hammett *σ*_p_^+^ values, a strong linear relationship can be observed (Fig. 4). Together with the excellent correlation observed when the experimentally obtained product selectivity is plotted against *σ*_p_^+^ (see Fig. 8 below), this data offer strong evidence for the significant influence of substrate electronic effect on charge polarization and consequent product selectivity.

**Table 1:** Computationally evaluated differences in charges between C^2^ and C^3^ centers for analogs of intermediate **3** bearing aziridine rings with different *p*-substituents

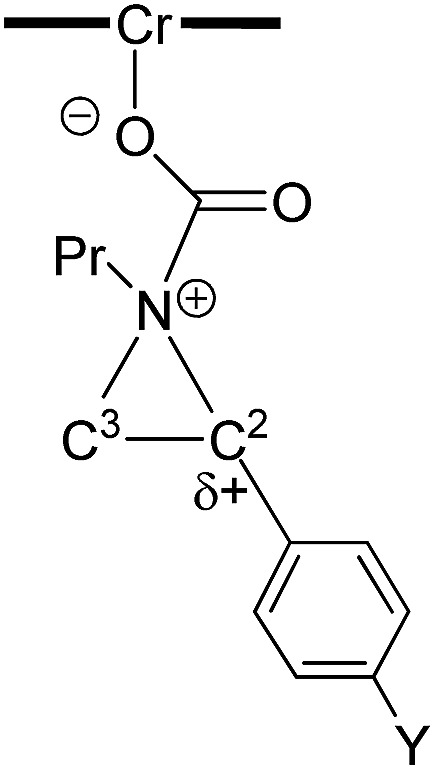			
Y	Charge at C^2^	Charge at C^3^	Difference in charge
OMe	0.122	–0.271	0.393
Me	0.107	–0.251	0.358
Cl	0.056	–0.238	0.294
H	0.049	–0.231	0.280
Br	0.040	–0.235	0.275

### Transition states (TS) calculations

**Fig. 5:**
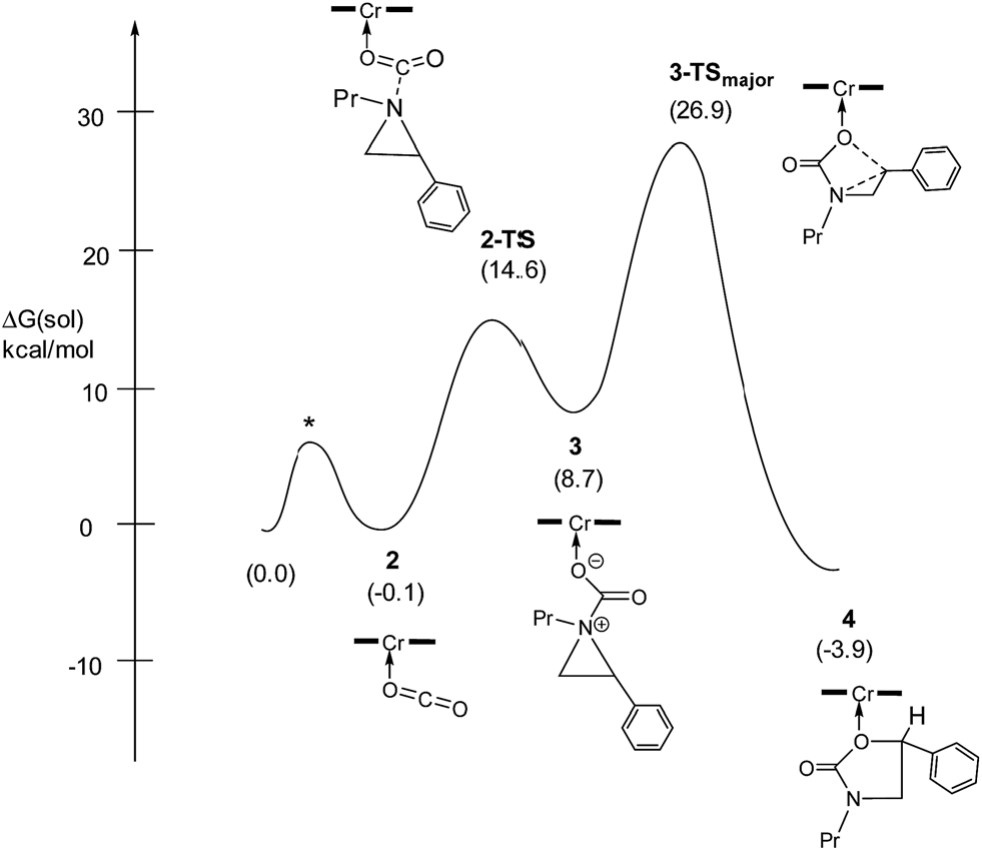
The complete free-energy profile for the formation of the major 5-substituted oxazolidinone product from the (salen)Cr^III^Cl-catalyzed [aziridine + CO_2_] coupling in the absence of DMAP cocatalyst. All energy values have been solvation-corrected. The TS marked as * was not located computationally and is only shown for illustrative purposes.

**Fig. 6:**
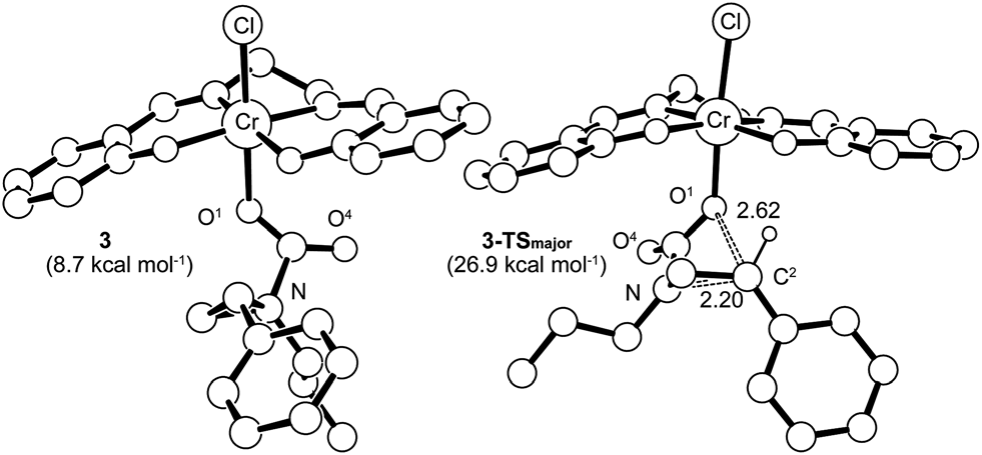
The **3-TS_major_** transition-state structure (right) for the formation of 5-substituted oxazolidinone, obtained through a synchronous and concerted pathway from intermediate **3** (left), where both C–N bond cleavage and C–O bond formation take place concomitantly. The “lengths” for both bonds (in Å) are indicated on the structure. For clarity, all of the hydrogens have been removed except for that on the C^2^ carbon where C–O bond formation is taking place.

Having verified that ring-opening is favored at the C^2^–N bond of intermediate **3** (Fig. 1), we located **3-TS_major_**, the alkoxide-mediated ring-opening transition state that leads to the 5-substituted oxazolidinone as the major product, at an energy of 26.9 kcal mol^–1^. As portrayed in the free energy profile for reaction 1 (Fig. 5), the ring-opening of the aziridine is likely the rate-determining step. The formation of **3-TS_major_** is *synchronous and concerted* in nature where both C–N bond cleavage and C–O bond formation take place at the same time, displaying elongated C···N and C···O bonds (2.20 Å and 2.62 Å, respectively, Fig. 6). Notably, this five-membered transition state structure adopts an envelope conformation that is typical of a five-membered ring. A close examination of this structure reveals that the phenyl substituent is in plane with the HC^2^–C^3^ group (the angle around the carbon bearing the –Ph group is 359.9°), effectively stabilizing the developing positive charge of the “incipient carbocation”. Indeed, the corresponding molecular orbital picture of **3-TS_major_** (ESI, Fig. S2†) shows that a substantial π-overlap exists between the phenyl substituent and the transiently carbocationic C^2^ carbon. This is consistent with an increase in the Mayer–Mulliken bond order (BO) for the C^2^–phenyl bond, to 1.29 from 1.02, that is considerably higher than that of a single bond (∼1.00). Upon free-geometry optimization, the located **3-TS_major_** easily leads to intermediate **4**, indicating a direct connection between it and the 5-substituted oxazolidinone product.

Interestingly, both the alkoxide and carbonyl oxygen in intermediate **3** (O^1^ and O^4^, respectively) are equally efficient for the subsequent nucleophilic aziridine ring-opening. The alternative ring-opening transition state **3-TS′_major_**, where O^4^ is the nucleophile, is electronically only 1.45 kcal mol^–1^ higher in energy than **3-TS_major_** and has essentially the same solvation-corrected free energy (see ESI, Fig. S3†).

It is important to note that the “*unimolecular*” aziridine ring-opening by a CO_2_-derived nucleophile is a unique feature in our proposed mechanism for reaction 1. In the reaction media that we employed (CH_2_Cl_2_ solvent) for this reaction, the amount of free chloride ion (or other alternative nucleophiles from the (salen)Cr^III^Cl catalyst) that can promote the ring-opening of any activated aziridine through a “*bimolecular*” mechanism would be negligible. The low level of chloride can be attributed to a combination of low catalyst loading (≤1 mol%) and the strong bond between the anionic chloride ligand and the cationic (salen)Cr^(III)^ center: our calculations suggest that the dissociation of chloride ion from (salen)Cr^III^Cl would cost a sizable energy penalty of 26.5 kcal mol^–1^. The catalyst, (salen)Cr^III^Cl itself, being a very poor nucleophile, also cannot open the activated aziridine ring in a bimetallic reaction mode (see details in ESI, section S6†).

**Fig. 7:**
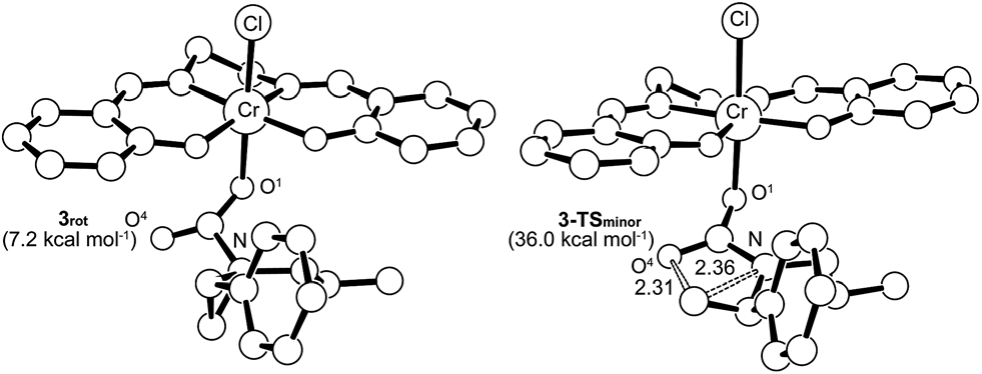
The transition state structure **3-TS_minor_** (right) for the formation of 4-substituted oxazolidinone, obtained through an asynchronous, concerted pathway form the corresponding intermediate **3_rot_** (left). The “lengths” for the relevant C–N and C–O bonds (in Å) are indicated on the structure. For clarity, all hydrogens have been removed.

Interestingly, transition-state searches for the path that leads to the 4-substituted oxazolidinone minor product revealed an *asynchronous*, *concerted* pathway to the **3-TS_minor_** transition state that is strictly based on the opening of the aziridine ring by the carbonyl functionality of **3** (Fig. 7, right structure). In contrast to **3-TS_major_**, the elongated C···N and C···O distances (2.36 Å and 2.31 Å, respectively) in this asynchronous TS are quite similar. In the gas phase, the electronic energy of **3-TS_minor_** was ∼7.3 kcal mol^–1^ higher than that of **3-TS_major_**, consistent with the disfavored formation for the 4-substituted oxazolidinone that was experimentally observed.^30^ Although the difference in solvation-corrected free energies between these two transition states (9.1 kcal mol^–1^) is higher than our expectation, it reproduces well the trend in favor of the major product.

**Fig. 8:**
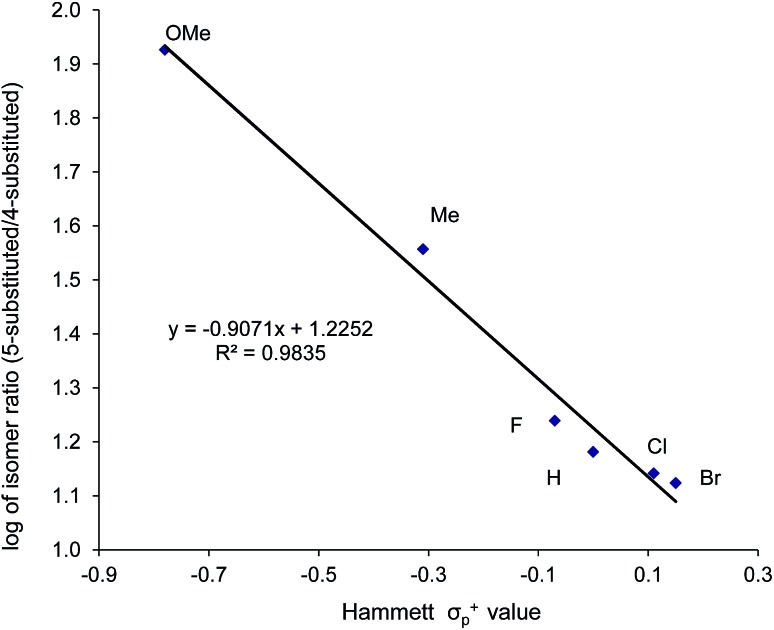
Experimental selectivity for 5-substituted oxazolidinone over the 4-substituted isomer as observed by Hammett competition experiment between *p*-substituted *N*-propyl-2-arylaziridines and the parent *N*-propyl-2-phenylaziridine. The selectivity decreases as the aryl group becomes more electron-withdrawing.

It is noteworthy that the pathway leading to **3-TS_minor_** starts with **3_rot_**, a rotamer of intermediate **3** where proper alignment of the respective interacting groups have been attained (Fig. 7). The required geometry is essentially isoenergetic to **3**. A closer scrutiny of **3-TS_minor_** discloses that the C=O bond is elongated considerably (from 1.21 to 1.24 Å), a direct consequence of its rehybridization into a nucleophilic C–O moiety. This change can be quantified by the change in the Mayer–Mulliken BO for the C=O group, which is reduced to 1.56 in **3-TS_minor_** from an initial value of 1.81 in **3_rot_**. Upon closer inspection, it becomes evident that the unstabilized incipient carbocation at C^3^ is so electron-deficient that the phenyl group on the adjacent C^2^ carbon is taking part in anchimeric assistance. As in the case of **3-TS_major_**, **3-TS_minor_** also adopts an envelope structure that is characteristic of five membered rings. Given the high energy of **3-TS_minor_**, we surmise that the small amount of 4-substituted oxazolidinone minor product observed under our experimental condition does not arise from the intramolecular opening of the aziridine ring by the carbonyl functionality of **3**. Instead, an alternative pathway may be operative where a (salen)Cr^III^-bound aziridine is opened by another aziridine molecule, in a manner similar to DMAP in the mechanism shown in Fig. 10. This intermediate then inserts CO_2_ and the minor product forms *via* ring-closing (see discussion below).

### Experimental selectivity for the (salen)Cr^III^Cl-catalyzed coupling of CO_2_ and aziridine in the absence of the DMAP cocatalyst

Thus far, our theoretical analyses predict a differential activation of the two carbons on the activated aziridine ring in intermediate **3** that eventually leads to the enhanced formation of **5**. Such differences in charge development should be apparent through product selectivity in a Hammett-type investigation. To this end, we examined the (salen)Cr^III^Cl-catalyzed coupling of CO_2_ with several *p*-substituted *N*-propyl-2-arylaziridines in the competitive presence of the parent *N*-propyl-2-phenylaziridine and in the absence of DMAP (eqn (2)). Notably, the experimental selectivity for the 5-substituted isomer was found to vary over almost an order of magnitude, with as high as 80 : 1 for *N*-propyl-2-(*p*-methoxyphenyl)aziridine and as low as 12 : 1 for *N*-propyl-2-(*p*-bromophenyl)aziridine.
2

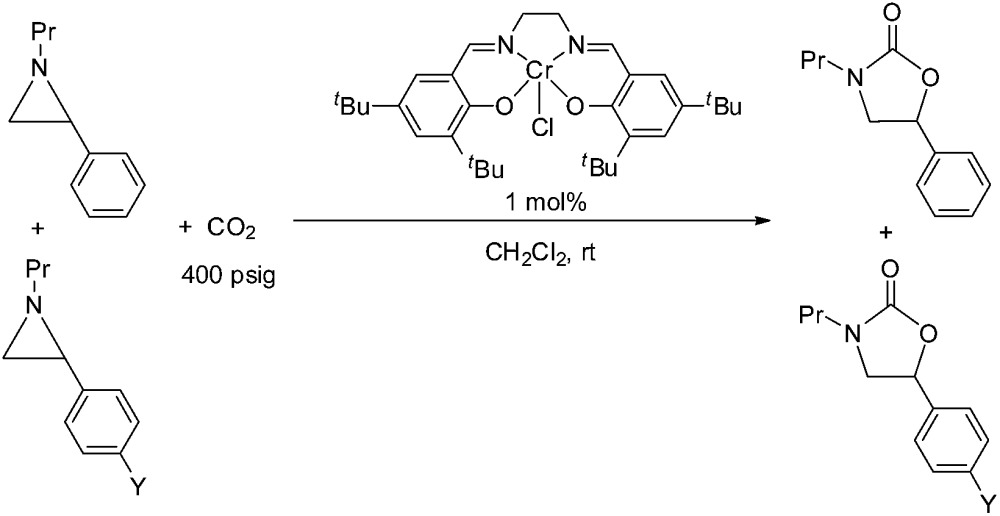




When plotted against the Hammett *σ*_p_^+^ values, the ratio of oxazolidinone products (5-substituted/4-substituted) obtained from the corresponding aziridines in reaction 2 afford an excellent linear correlation (*R*^2^ = 0.98), signifying a clear influence of substrate electronic effects on product selectivity. In addition, the moderate magnitude of this negative *ρ* (–1.28) is consistent with the presence of an incipient cationic character^40^ at the aryl-bearing C^2^ in the aziridine ring-opening step. This fits well with our computational results that the aziridine N–C^2^ bond becomes polarized upon attacking the coordinated CO_2_ moiety on the [(salen)Cr^III^Cl center. That the trend of experimentally observed selectivity closely mirrors the computationally evaluated charge-separation correlation (Fig. 4) greatly strengthens our proposed mechanism (Fig. 1). We note that plotting the experimental selectivity ratio *vs.* Hammett *σ*_p_ and *σ*_p_^–^ gave only poor correlations, further confirming the cationic nature of the aryl-bearing C^2^ carbon.

**Fig. 9:**
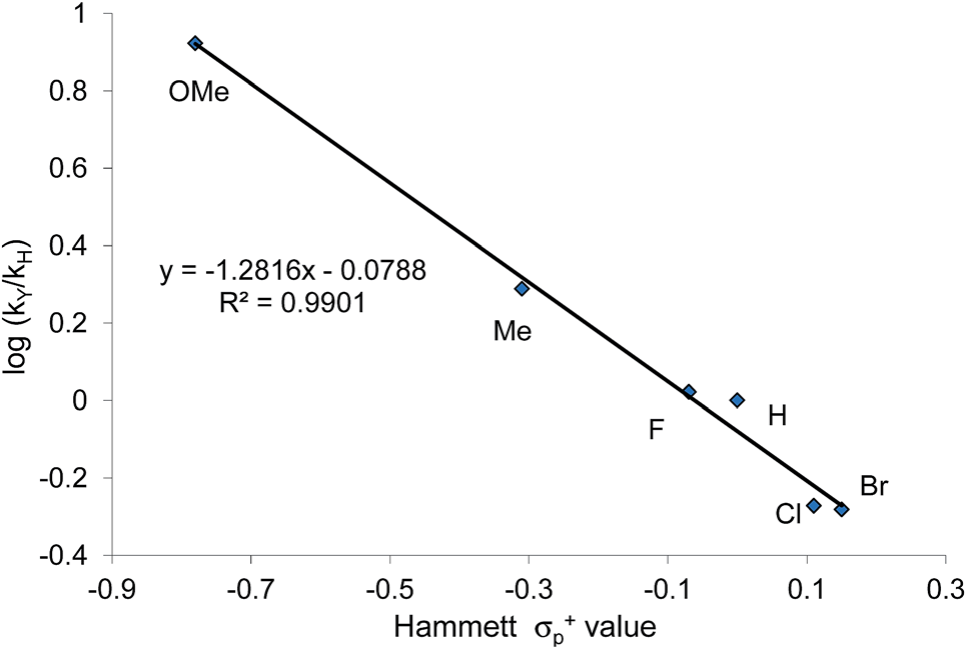
A Hammett correlation of reaction rates for the *N*-propyl-2-arylaziridine substrate (relative to the parent *N*-propyl-2-phenylaziridine) shown in reaction 2 against *σ*_p_^+^ values. A linear decrease is observed as more electron-withdrawing substituents are placed on the 2-aryl group of the *N*-propyl-2-arylaziridine substrate. Relative reaction rate constants are obtained against the rate for *N*-propyl-2-phenyl aziridine.

That the two carbons in the complexed 2-aryl-substituted aziridine in **3** are differently activated suggests that the rate of the oxazolidinone formation in reaction 1 will also be influenced by the presence of electron-donating and -withdrawing groups at the *p*-position of the aryl ring in the aziridine substrate. Stabilization of the incipient carbocation by electron-donating groups will accelerate the rate of oxazolidinone formation while the presence of electron-withdrawing group will retard this rate. Indeed, a plot of the relative rate constants for reaction 2 against Hammett *σ*_p_^+^ values clearly shows a linear relationship with more electron-withdrawing groups affording lower reaction rates (Fig. 9). These data further substantiate our mechanistic proposal that the (salen)Cr^III^Cl-catalyzed [aziridine + CO_2_] coupling proceeds through intermediates bearing incipient cationic charges.

### The effect of DMAP on isomer selectivity

**Fig. 10:**
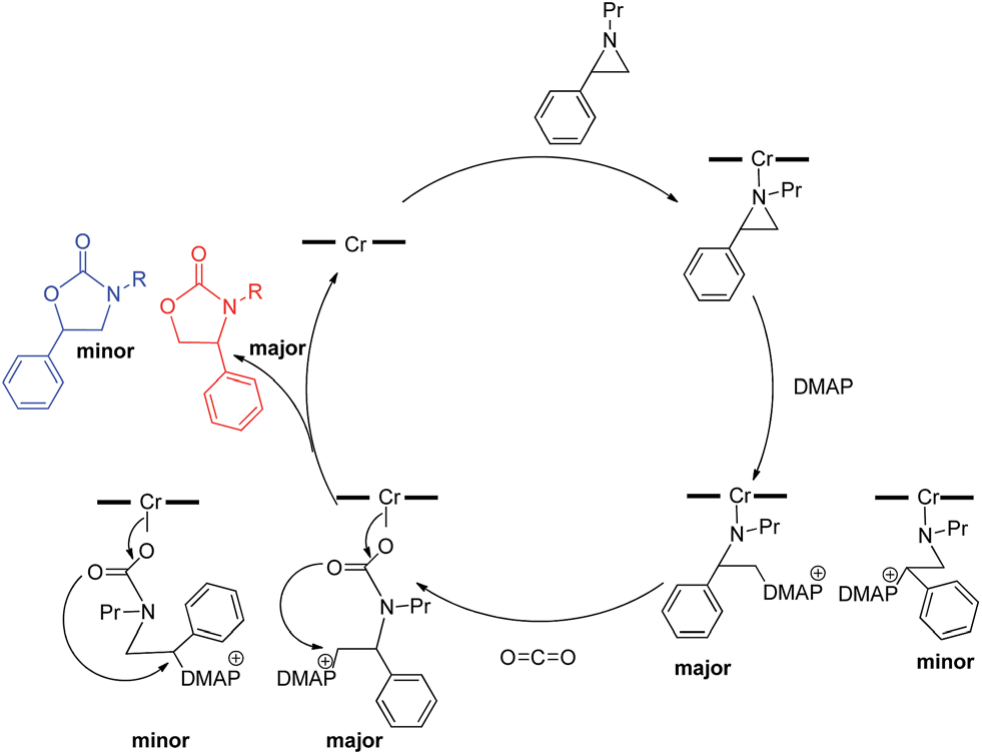
A plausible mechanism through which the erosion of selectivity in the presence of DMAP can be explained.

**Fig. 11:**
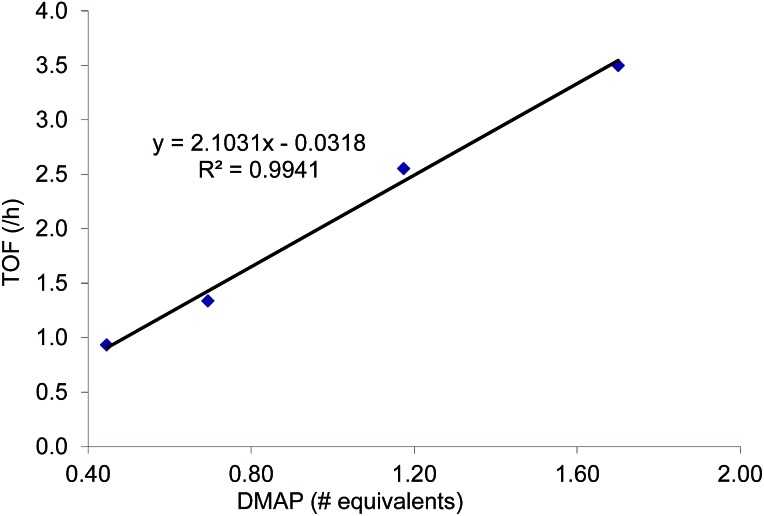
The variation in rate for the coupling reaction between *N*-propyl-2-phenylaziridine and CO_2_ catalyzed by (salen)Cr^III^Cl in the presence of varying amounts of DMAP cocatalyst (eqn (3)). Reaction conditions: *N*-propyl-2-phenylaziridine (0.322 g, 2 mmol), (salen)Cr^III^Cl catalyst (12.6 mg, 0.02 mmol), DMAP cocatalyst (varying amounts: 0.45 to 1.70 equiv. with respect to catalyst), 400 psig CO_2_, CH_2_Cl_2_ (3.7 mL), rt, 24 h.

**Fig. 12:**
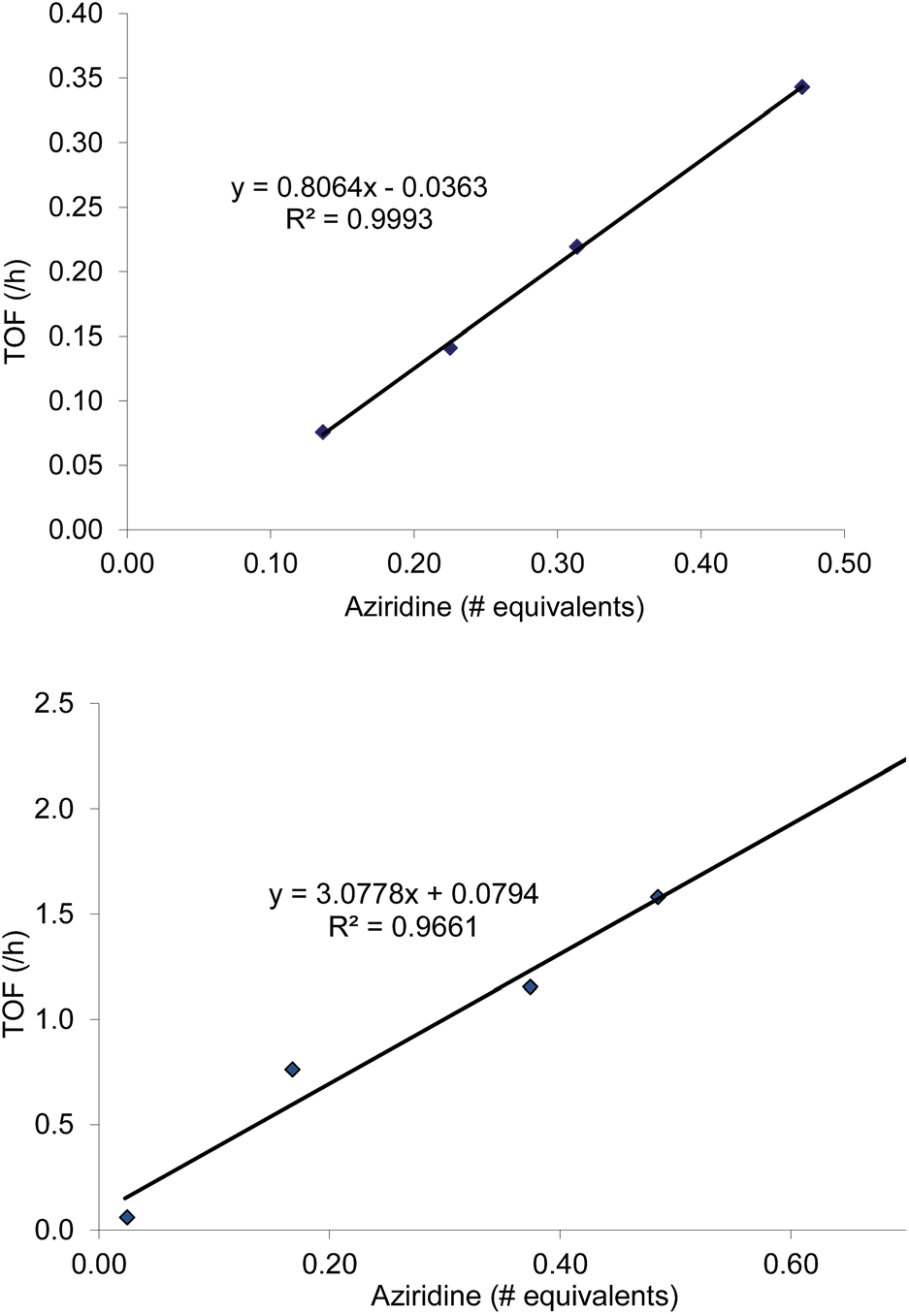
Kinetic plots of the coupling reaction between *N*-propyl-2-phenylaziridine and CO_2_ catalyzed by (salen)Cr^III^Cl. General reaction conditions: *N*-^*n*^propyl-2-phenylaziridine (varying amounts, between 0.088 g and 0.228 g; 0.55 mmol to 1.42 mmol), catalyst (5.8 mg, 0.01 mmol), 400 psig CO_2_, CH_2_Cl_2_ (1.8 mL), rt, 24 h. Top: in the absence of DMAP cocatalyst. Bottom: in the presence of 2 equiv. of DMAP cocatalyst (2.45 mg, 0.02 mmol).

As mentioned in the introduction, the rate of reaction 1 is slowed down in the absence of the DMAP cocatalyst but afford higher selectivity, up to 80 : 1 for the *N*-propyl-2-(*p*-methoxyphenyl)aziridine substrate. Adding a Lewis-basic cocatalyst enhances the rate but compromises selectivity, suggesting the presence of another mechanistic pathway. Such a decrease in selectivity can be explained if the aziridine coordinates to the (salen)Cr center first and is then activated for ring-opening by DMAP (Fig. 10). In this case, the sterically driven preference for ring-opening will be the opposite of that shown in Fig. 1: DMAP would prefer to attack the coordinated aziridine at the less-substituted C^3^. CO_2_ insertion into the Cr–N bond followed by ring-closing to displace the DMAP leaving group will yield intermediates **4**, but with opposite preference from that shown in Fig. 1, thus eroding the excellent selectivity (favoring the 5-substituted isomer) observed for the DMAP-free reaction. Such a process would have linear dependences on the concentrations of both DMAP and aziridine. This is indeed the case: the rate for reaction 3 exhibits single-order rate dependence on the concentration of DMAP (Fig. 11), consistent with its role as a ring-opening nucleophile. In addition, it exhibits single-order rate dependence on the concentration of the aziridine substrate, both in the absence and presence of the DMAP cocatalyst (Fig. 12).
3

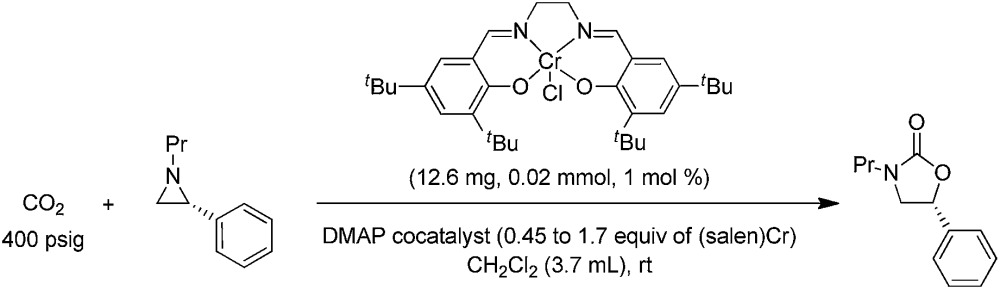




### Experimental support for an incipient carbocation intermediate: retention of chirality by chiral aziridine substrates

Because reaction 1 passes through a concerted, synchronous transition state (*i.e.*, **3-TS_major_**), a true carbocation intermediate does not exist; rather, an incipient carbocation is more probable. As such, a chiral aziridine undergoing coupling with CO_2_ should retain the stereochemistry at its chiral carbon under our reaction conditions. Indeed, treatment of (*R*)-*N*-propyl-2-phenylaziridine with CO_2_ in the presence of the achiral catalyst **1** resulted in no racemization for either the substrate or product. Additionally, the products of the coupling between CO_2_ and pure samples of either *cis*- or *trans-N*-propyl 2,3-dipropylaziridine retain their respective diastereopurities (eqn (4) and (5)). Together, these data support our argument that an incipient carbocation intermediate is more likely than a true carbocation, whose presence would most likely lead to loss of chirality as recently observed by Wender and others in a Ag^+^-catalyzed [aziridine + alkyne] coupling.^40^–^42^

4

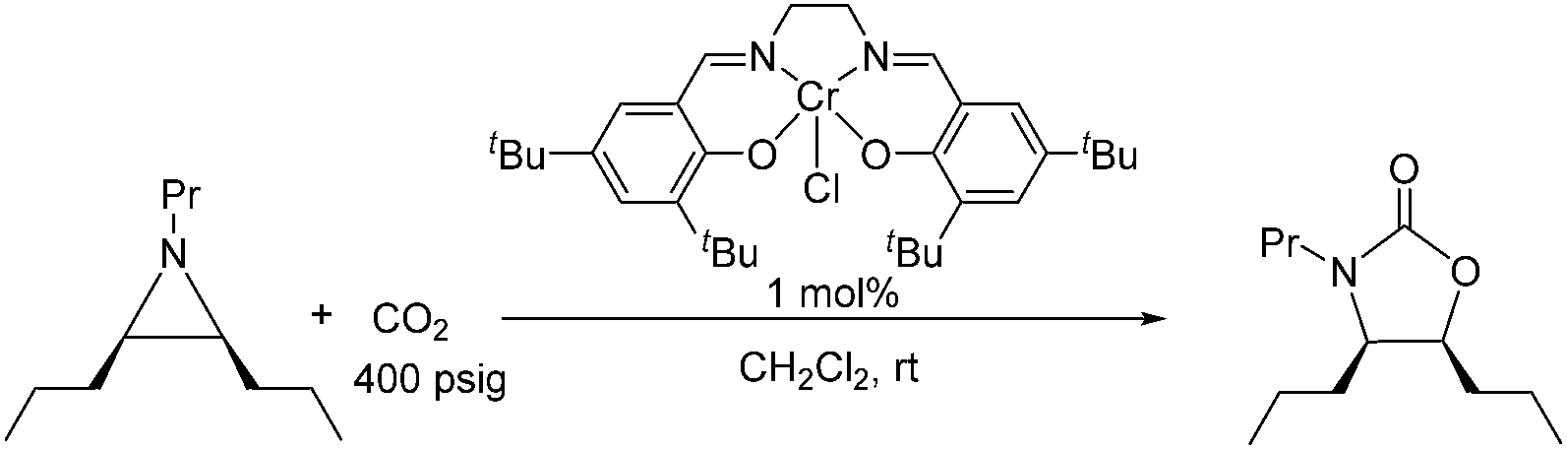



5

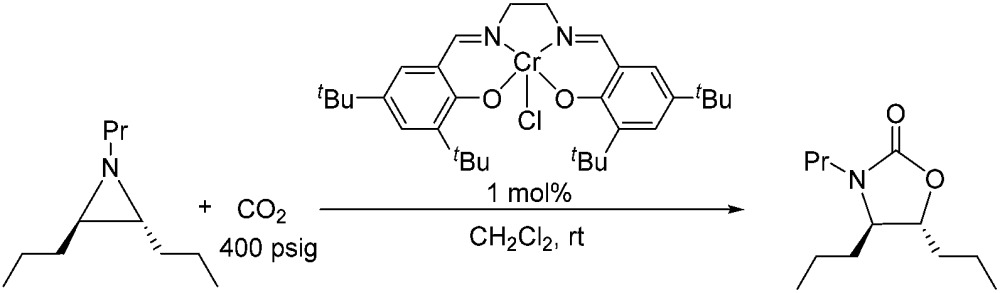




## Conclusions

In summary, we presented the complete mechanism of the (salen)Cr^III^Cl-catalyzed [aziridine + CO_2_] coupling to form 5-substituted oxazolidinones in a highly selective fashion, which is quite unique among the known catalytic methods for coupling aziridine and CO_2_. Through a combined theoretical and experimental study of the mechanism of this coupling reaction, we were able to attribute this distinctive selectivity to the preferential *intramolecular* ring-opening at the more substituted carbon of the aziridine ring in a (salen)Cr^III^(aziridiumcarbamate) intermediate. Theoretical modeling and transition state search reveal that such a process is only possible through an initial key coordination of the CO_2_ molecules to the (salen)Cr^III^ center, which activates the CO_2_ carbon for nucleophilic attack by the aziridine substrate. In the resulting (salen)Cr^III^(aziridiumcarbamate) intermediate, either of the two carbamate oxygen atoms can act as an *intramolecular* nucleophile to ring-open the aziridine moiety, allowing for an exquisite control of the regioselectivity. Together, these results also shed light on the erosion of selectivity for the 5-substituted isomer when the (salen)Cr^III^Cl-catalyzed [aziridine + CO_2_] coupling is carried out in the presence of the DMAP cocatalyst.

Notably, we showed through a detailed Hammett study that while there is not a significant formal charge development in the aforementioned ring-opening process, its intramolecular nature and the incipient cationic nature of the aziridine C^2^ allows for the substituents of the aziridine substrates to have a substantial influence on both their reactivities with CO_2_ and the selectivities for the final product. Indeed, the broad range of selectivity for 5- *vs.* 4-substituted oxazolidinone varies almost over an order of magnitude for different aziridines (80 : 1 for *N*-propyl-2-(*p*-methoxyphenyl)aziridine to 12 : 1 for *N*-propyl-2-(*p*-bromophenyl)aziridine).

## Supplementary Material

Supplementary information
