# Supplementary material for: Intramolecular ring-opening from a CO2-derived nucleophile as the origin of selectivity for 5-substituted oxazolidinone from the (salen)Cr-catalyzed [aziridine + CO2] coupling
Source: Chem Sci. 2014 Nov 21;6(2):1293–300. doi: 10.1039/c4sc02785j (PMC5811137; doi:10.1039/c4sc02785j)
Supplement: Supplementary file 1 [file SC-006-C4SC02785J-s001.pdf]

Electronic Supplementary Material (ESI) for Chemical Science.
This journal is © The Royal Society of Chemistry 2014

[Electronic Supplementary Information to accompany *Chem. Sci.* MS# SC-EDG-09-2014-002785]

Intramolecular ring-opening from a CO_2-derived nucleophile as the origin of selectivity for 5-substituted oxazolidinone from the (salen)Cr-catalyzed [aziridine + CO_2] coupling

Debashis Adhikari,[a,b] Aaron W. Miller,[a] Mu-Hyun Baik,[b,c,]* SonBinh T. Nguyen[a,]*
[a]*Department of Chemistry and the Institute for Catalysis in Energy Processes, Northwestern University, 2145 Sheridan Road, Evanston, Illinois 60208-3113, USA,* [b]*Department of Chemistry, Indiana University, 800 East Kirkwood Avenue, Bloomington, IN 47405 (USA),* [c]*Department of Materials Chemistry, Korea University, Jochiwon-eup, Sejong-si, 339-700, South Korea*
[*]*Email: mbaik@indiana.edu (M.-H.B) and stn@northwestern.edu (S.T.N.)*

Table of Contents *Page number*

S1. Computational details *S1*
S2. General procedures and materials *S2*
S3. General experimental procedure for obtaining Hammett data *S2*
S4. Computational evaluation of the binding of aziridine to the (salen)Cr^{III}Cl center *S2*
S5. Correction factors for the free energy calculations *S2*
S6. Results of attempts to geometrically optimize intermediates in potential bimetallic mechanisms *S4*
S7. Characterization data for the oxazolidinone products *S4*
S8. Coordinates and vibrational frequencies of investigated structures *S5*
S9. Authors contributions audit *S11*
S10. References *S12*

S1. Computational details

All calculations were carried out using Density Functional Theory as implemented in the Jaguar 7.8 suite[S1] of *ab initio* quantum chemistry programs. Geometry optimizations were performed using the M06 functional[S2] and the 6-31G** basis set. Chromium was represented using the Los Alamos LACVP basis[S3, S4] that includes relativistic effective core potentials. The energies of the optimized structures were reevaluated by additional single-point energy calculations of each optimized geometry using Dunning's correlation consistent triple-ζ basis set,[S5] cc-pVTZ(-f) that includes a double set of polarization functions. All stationary points were verified to be minima or transition states by proper vibrational analysis at the double- ζ level.

Solvation calculations were carried out with the 6-31G**/LACVP basis at the optimized gas-phase geometry employing a dielectric constants of $\varepsilon = 9.08$ for dichloromethane. Solvation energies were evaluated using a self-consistent reaction field (SCRF) approach based on accurate numerical solutions of the Poisson-Boltzmann equation.[S6-S8] For all continuum models, the solvation energies are subjected to empirical parameterization of the atomic radii that are used to generate the solute surface. We employed the standard set[S9] of optimized radii in Jaguar for H (1.150 Å), C (1.900 Å), O (1.600 Å), N (1.600 Å) and Cr (1.511Å). Analytical vibrational frequencies within the harmonic approximation were computed with the 6-31G**/LACVP basis set to confirm proper convergence to well-defined minima or saddle points on the potential energy surface. The free energy of a molecule in solution phase, G(Sol), is computed as follows:

$$G(\text{Sol}) = G(\text{gas}) + G^{\text{solv}} \quad \text{(S2)}$$

$$G(\text{gas}) = H(\text{gas}) - TS(\text{gas}) \quad \text{(S3)}$$

$$H(\text{gas}) = E(\text{SCF}) + ZPE \quad \text{(S4)}$$

$$\Delta G(\text{Sol}) = \sum G(\text{Sol}) \text{ for products} - \sum G(\text{Sol}) \text{ for reactants} \quad \text{(S5)}$$

where G(gas) is the free energy of the molecule in the gas phase; G^{solv} is the free energy of solvation as computed using the continuum solvation model; H(gas) and S(gas) are the enthalpic and entropic components of the molecule in the gas phase, respectively; T is the temperature (298.15 K); E(SCF) is the self-consistent field energy, i.e., the "raw" electronic energy as computed from the SCF procedure; and ZPE is the zero point energy. The energy of the species in the potential energy surface diagram has been reported after taking account of the concentration effect for excess aziridine and carbon di oxide in the reaction medium.

To locate transition states, the potential energy surface was first explored approximately using the linear synchronous transit (LST) method,[S10] followed by a quadratic synchronous transit (QST)[S11] search that uses the LST transition state as an initial guess. In QST, the initial part of the transition state search is restricted to a circular

curve connecting the reactant, initial transition state guess, and the product, followed by a search along the Hessian eigenvectors tangential to this curve. In the relative energy diagram, the ΔG(Sol) values have been corrected to account for the excess concentrations of aziridine and high CO_2 pressure. To decrease the computational cost, the *tert*-Bu groups of the salen ligand were replaced with hydrogens and all the calculations were carried out with this truncated framework. We assume that such a small change in the steric environment would not significantly change the electronic environment of the (salen)Cr^{III} complex. As this complex can potentially be in both doublet and quartet spin configurations,[S12] we initially performed several calculations on both of these states. In all cases, the quartet configuration was observed to give the lowest ground-state energy, and was used in all subsequent calculations.

S2. General procedures and materials

^{1}H and ^{13}C NMR spectra were recorded on a Varian Inova 500 (499.570 MHz for ^{1}H, 125.631 MHz for ^{13}C) spectrometer. NMR data are reported as follows: chemical shift (multiplicity (b = broad, s = singlet, d = doublet, t = triplet, q = quartet, and m = multiplet), and integration. ^{1}H and ^{13}C chemical shifts are reported in ppm downfield from tetramethylsilane (TMS, δ scale) with the solvent resonances as internal standards. Assignment of NMR spectra are made using ACD Labs software version 4.02 (Advanced Chemistry Development, Inc., Toronto, Canada).

IR spectra were collected on a Nicolet 5PC instrument and analyzed using Nicolet's PC-IR software. Elemental analyses were provided by Atlantic Microlab, Inc. (Norcross, GA).

GC-MS experiments were carried out on an Agilent Technologies 6890N gas chromatograph-mass spectrometer equipped with a 30-m HP-5 capillary column (0.32-mm inner diameter and 0.25-mm film thickness) and Agilent's MSD ChemStation software version D.01.02 build 16. Temperature program: intitial time = 2 min, initial temperature = 60 °C, rate = 20 °C/min; final time = 12 min, final temperature = 250 °C. GC analyses of oxazolidinone products were carried out on a Hewlett-Packard 5890A gas chromatograph equipped with an FID detector and a 30-m HP-5 capillary column (0.32-mm inner diameter and 0.25-mm film thickness). Temperature program: initial time = 0 min, initial temperature = 60 °C, rate = 20 °C/min; final time = 2.5 min, final temperature = 250 °C.

Catalyst **1**[S13, S14] and the aziridine precursors were synthesized according to published procedures.[S15] Synthetic reagents (Aldrich Chemical), solvents for the catalytic reactions (Fischer Chemical or Aldrich Chemical), and deuterated solvents (Cambridge Isotope Laboratories) were obtained from commercial sources and used as received. Diethyl ether for the IR study was dried over activated alumina and Q5 catalysts using the Dow-Grubbs solvent system installed by Glass Contours, Inc. (now J. C. Meyer Solvent System, Laguna Beach, CA). Except for *N*-^{n}propyl-5-phenyloxazolidinone the remaining oxazolidinones have not been made and their analytical data are reported herein.

S3. General experimental procedure for obtaining Hammett and kinetic data

Hammett data. On the bench top, a 45 mL Parr high-pressure reactor equipped with a magnetic stir bar was charged with catalyst **1** (12.6 mg, 2×10^{-5} mol). A *p*-substituted *N*-^{n}propyl-2-phenylaziridine (2 mmol) was then taken up in a gas-tight syringe, followed by enough CH_2Cl_2 (approximately 0.7 mL) to fill the 1 mL volume in the syringe, and added to the reactor. The syringe was subsequently rinsed with CH_2Cl_2 (1 mL), and the rinse was also added to the reactor to ensure complete loading of all of the substrate. This process was repeated with *N*-^{n}propyl-2-phenylaziridine (0.322 g, 2 mmol) to give a final 4 mL solution of both aziridines in CH_2Cl_2 (0.5 M solution in each aziridine). Finally, undecane (100 μL, 0.474 mmol, internal standard) was placed in the reactor.

The reactor assembly was subsequently sealed and placed under constant CO_2 pressure (400 psig) for 10 minutes to allow for equilibration. The CO_2 valve was then closed, and the reactor was placed on a magnetic stirring plate. After a desired period, the reactor was carefully vented inside a fume hood and a small aliquot was then removed from the solution for GC analysis. The catalyst was removed from this aliquot by elution with CH_2Cl_2 (20 mL) through a wet-packed silica plug (15 mm × 5 mm), which was prepared with 5 vol % triethylamine in CH_2Cl_2. Reactivity ratio was determined via GC using peak areas against undecane internal standard.

Kinetic data. For the kinetic experiments, the same experimental set-up and data sampling described above was used except that only *N*-^{n}propyl-2-phenylaziridine was used as the substrate. The concentrations of reactants and reaction conditions are provided in the captions of the figures in the main manuscript.

S4. Computational evaluation of the binding of aziridine to the (salen)Cr^{III}Cl center

The binding of *N*-^{n}propyl-2-phenylaziridine to the Lewis-acidic (salen)Cr^{III}Cl center will activate the aziridine ring, as computationally observed through the elongation of the C–N bonds (Figure S1). But this activation is not sufficient to spontaneously open the ring. Computationally, even if we start with a geometry where the ring is initially opened, geometrical optimization will eventually convert it back to the ring-closed structure

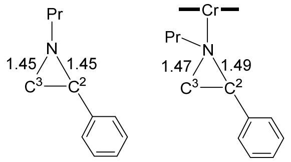

Figure S1. The elongation of the C–N bonds in *N*-^{n}propyl-2-phenylaziridine upon binding to the Lewis-acidic (salen)Cr^{III} center. Shown are the bond lengths (Å) for the free and activated aziridine when bound to (salen)Cr^{III}. The preferential elongation of C^2–N is quite clear when being compared to C^3–N.

S5. Correction factors for the free energy calculations

In our experimental conditions, the aziridine concentration was 100 times more than that of the (salen)Cr^{III}Cl catalyst. To account for such a significantly high concentration effect, a correction factor of 2.73 kcal mol^{-1} (at 25 ^{o}C by using the equation $\Delta G = -RT\ln K$, where K is the ratio of concentrations of the substrate and the catalyst) has been included in the computation. The dissolved concentration of CO_2 in dichloromethane[S16] was also similarly included to give a correction factor of 3.84 kcal mol^{-1}.

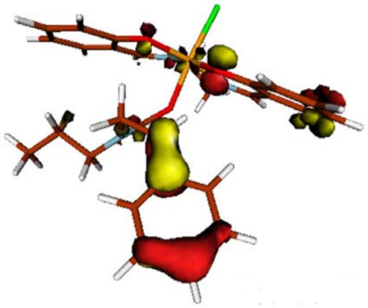

Figure S2. The HOMO-21 orbital of **3-TS$_{major}$** showing the π-conjugation of the substituent phenyl group with transient carbocation.

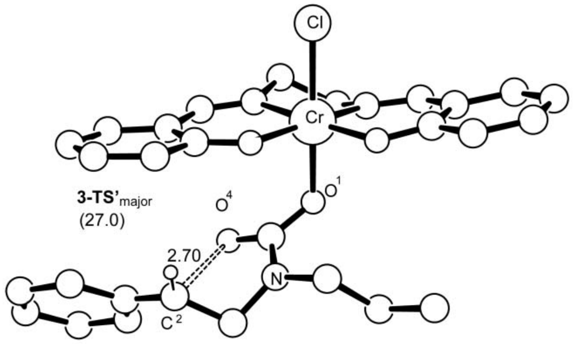

Figure S3. The computed structure of the alternative transition state **3-TS'$_{major}$**, where the carbonyl oxygen (O) is behaving as a nucleophile en route to the formation of the 5-substituted oxazolidinone product. This transition state is concerted and asynchronous in nature. For clarity, all of the hydrogens have been removed except for that on the C^2 carbon where the C^2–O^4 bond formation is taking place.

S6. Results of attempts to geometrically optimize intermediates in potential bimetallic mechanisms

We also attempted to computationally evaluate the possibility of (salen)$Cr^{III}Cl$ being able to promote the ring-opening of an activated aziridine in a bimetallic fashion.[S17] Unfortunately, multiple attempts of geometry optimization either failed to converge after many iterations or led to chemically unreasonable structures. For example, the Cr–Cl bond of the nucleophilic (salen)$Cr^{III}Cl$ in one of the optimized intermediate structures was stretched to a very long distance of 3.47 Å. When the geometry optimizations were carried out keeping the C–Cl distance (carbon of the Lewis-acid activated aziridine ring which requires opening and Cl from another (salen)$Cr^{III}Cl$) constrained to a reasonable value to help the aziridine ring-opening, it was observed that that chloride ligand detached completely from the Cr^{III} center. Lastly, when the starting-guess geometry was varied to contain a closed aziridine ring with an appropriately aligned Cr–Cl bond to facilitate the ring-opening, the geometry optimization did not produce a ring-opened product.

S7. Characterization data for the oxazolidinone products

***N*-*^n^*propyl-5-(*p*-tolyl)oxazolidinone**. A clear liquid. ^{1}H NMR ($CDCl_3$, 500 MHz): δ 0.95 (t, 3H, propyl C*H*$_3$, ^3J = 7.0 Hz), 1.60 (m, 2H, propyl C*H*$_2$), 2.37 (s, 3H, methyl C*H*$_3$), 3.28 (m, 2H, propyl N-C*H*$_2$), 3.43 (t, 1H, N-C*H*$_2$, ^3J = 8.0 Hz), 3.90 (t, 1H, N-C*H*$_2$, ^3J = 8.5 Hz), 5.46 (t, 1H, O-C*H*, ^3J = 8.0 Hz), 7.24 (m, 5H, arom-*H*). ^{13}C{^{1}H}NMR ($CDCl_3$, 125 MHz): δ 11.3 (propyl *C*H$_3$), 20.9 (propyl *C*H$_2$), 21.4 (tosyl *C*H$_3$), 46.1 (propyl *C*H$_2$), 52.4 (N-*C*H$_2$), 74.6 (O-*C*H), 125.8 (C_m), 129.8 (C_o), 136.1 (C_p), 138.9 (C_i), 158.3 (*C*=O). FTIR (thin film): ν_{CO} 1753 cm^{-1}. Anal.: Calcd. for $C_{13}H_{17}NO_2$: C, 71.21; H, 7.81; N, 6.39; Found: C, 71.24, H, 7.85, N, 6.38. GC-MS(EI): Calcd. for $C_{13}H_{17}NO_2$: 219.1, Found: 219.2.

***N*-*^n^*propyl-5-(*p*-methoxyphenyl)oxazolidinone**. A clear liquid. ^{1}H NMR ($CDCl_3$, 500 MHz): δ 0.95 (t, 3H, propyl C*H*$_3$, ^3J = 7.0 Hz), 1.59 (m, 2H, propyl C*H*$_2$), 3.28 (m, 2H, propyl N-C*H*$_2$), 3.45 (t, 1H, N-C*H*$_2$, ^3J = 8.0 Hz), 3.73 (s, 3H, OC*H*$_3$), 3.92 (t, 1H, N-C*H*$_2$, ^3J = 8.5 Hz), 5.44 (t, 1H, O-C*H*, ^3J = 8.0 Hz), 7.20 (m, 5H, arom-*H*). ^{13}C{^{1}H} NMR ($CDCl_3$, 125 MHz): δ 11.3 (propyl *C*H$_3$), 20.9 (propyl *C*H$_2$), 46.1 (propyl *C*H$_2$), 52.4 (N-*C*H$_2$), 55.1 (methoxy *C*H$_3$), 75.2 (O-*C*H), 112.7 (C_m), 127.4 (C_o), 131.1 (C_i), 156.4 (*C*=O), 158.7 (C_p). FTIR (thin film): ν_{CO} 1756 cm^{-1}. Anal.: Calcd. for $C_{13}H_{17}NO_3$: C, 66.36; H, 7.28; N, 5.95; Found: C, 66.43, H, 7.03, N, 5.78. GC-MS(EI): Calcd. for $C_{13}H_{17}NO_3$: 235.3, Found: 235.2.

***N*-*^n^*propyl-5-(*p*-chlorophenyl)oxazolidinone**. A clear liquid. ^{1}H NMR ($CDCl_3$, 500 MHz): δ 0.95 (t, 3H, propyl C*H*$_3$, ^3J = 7.0 Hz), 1.59 (m, 2H, propyl C*H*$_2$), 3.28 (m, 2H, propyl N-C*H*$_2$), 3.40 (t, 1H, N-C*H*$_2$, ^3J = 8.0 Hz), 3.93 (t, 1H, N-C*H*$_2$, ^3J = 8.5 Hz), 5.48 (t, 1H, O-C*H*, ^3J = 8.0 Hz), 7.35 (m, 5H, arom-*H*). ^{13}C{^{1}H} NMR ($CDCl_3$, 125 MHz): δ 11.3 (propyl *C*H$_3$), 20.9 (propyl *C*H$_2$), 46.1 (propyl *C*H$_2$), 52.3 (N-*C*H$_2$), 73.8 (O-*C*H), 127.1 (C_m), 129.4 (C_o), 134.9 (C_p), 137.7 (C_i), 158.0 (*C*=O). FTIR (thin film): ν_{CO} 1753 cm^{-1}. Anal.: Calcd. for $C_{12}H_{14}ClNO_2$: C, 60.13; H, 5.89; N, 5.84; Cl, 14.79; Found: C, 60.33; H, 5.88; N, 5.78; Cl, 14.84. GC-MS(EI): Calcd. for $C_{12}H_{14}ClNO_2$: 239.1, Found: 239.2.

***N*-*^n^*propyl-5-(*p*-fluorophenyl)oxazolidinone**. A clear liquid. ^{1}H NMR ($CDCl_3$, 500 MHz): δ 0.94 (t, 3H, propyl C*H*$_3$, ^3J = 7.0 Hz), 1.59 (m, 2H, propyl C*H*$_2$), 3.28 (m, 2H, propyl N-C*H*$_2$), 3.40 (t, 1H, N-C*H*$_2$, ^3J = 8.0 Hz), 3.90 (t, 1H, N-C*H*$_2$, ^3J = 8.5 Hz), 5.48 (t, 1H, O-C*H*, ^3J = 8.0 Hz), 7.20 (m, 5H, arom-*H*). ^{13}C{^{1}H} NMR ($CDCl_3$, 125 MHz): δ 11.3 (propyl *C*H$_3$), 20.9 (propyl *C*H$_2$), 46.0 (propyl *C*H$_2$), 52.4 (N-*C*H$_2$), 74.0 (O-*C*H), 116.1 (C_m, J = 21.5 Hz), 127.7 (C_o), 134.9 (C_p), 158.0 (*C*=O), 163.0 (C_i, J = 253.5 Hz),. FTIR (thin film): ν_{CO} 1751 cm^{-1}. Anal.: Calcd. for $C_{12}H_{14}FNO_2$: C, 64.56; H, 6.32; N, 6.27; F, 8.51; Found: C, 64.57; H, 6.31; N, 6.27; F, 8.41. GC-MS(EI): Calcd. for $C_{12}H_{14}FNO_2$: 223.1, Found: 223.2.

***N*-*^n^*propyl-5-(*p*-bromophenyl)oxazolidinone**. A clear liquid. ^{1}H NMR ($CDCl_3$, 500 MHz): δ 0.95 (t, 3H, propyl C*H*$_3$, ^3J = 7.0 Hz), 1.59 (m, 2H, propyl C*H*$_2$), 3.28 (m, 2H, propyl N-C*H*$_2$), 3.39 (t, 1H, N-C*H*$_2$, ^3J = 8.0 Hz), 3.93 (t, 1H, N-C*H*$_2$, ^3J = 8.5 Hz), 5.47 (t, 1H, O-C*H*, ^3J = 8.0 Hz), 7.41 (m, 4H, arom-*H*). ^{13}C{^{1}H} NMR ($CDCl_3$, 125 MHz): 127.4 (C_m), 132.3 (C_o), 138.2 (C_p), 138.2 (C_i), 158.0 (*C*=O). FTIR (thin film): ν_{CO} 1750 cm^{-1}. Anal.: Calcd. for $C_{12}H_{14}BrNO_2$: C, 50.72; H, 4.97; N, 4.93; Br, 28.12; Found: C, 50.75; H, 4.96; N, 4.94; Br, 28.13. GC-MS(EI): Calcd. for $C_{12}H_{14}BrNO_2$: 283.0, Found: 283.1

S8. Coordinates and vibrational frequencies of investigated structures

Table S1. Cartesian coordinates

==================================

1

==================================

Cr	2.170169301	0.852481541	0.347487018
Cl	3.612991178	2.160351859	1.440406410
N	2.301389360	-0.649208299	1.692443847
C	1.671035457	-0.290827125	2.954881037
C	0.356862512	0.402829220	2.625689540
N	0.591085276	1.323170103	1.516296899
C	-0.153156874	2.370525378	1.358297568
C	-0.094820713	3.277806653	0.261599490
C	0.732013652	3.040312326	-0.882176438
C	0.633513877	3.954641056	-1.957131445
C	-0.212788449	5.037946001	-1.897073488
C	-1.019905080	5.275118291	-0.769616249
C	-0.956722278	4.396381811	0.283906636
O	1.527485181	2.022311602	-0.999921942
C	2.921304929	-1.772702654	1.537973692
C	3.595581800	-2.193524099	0.353761273
C	4.191996983	-3.472764262	0.352144307
C	4.826565362	-3.968221444	-0.761893683
C	4.875541095	-3.172939640	-1.919902331
C	4.307216675	-1.919169460	-1.952550336
C	3.649594771	-1.381498720	-0.822141651
O	3.110946084	-0.203091697	-0.917239625
H	-0.053321924	0.924486514	3.502199524
H	1.510272602	-1.170452646	3.595018839
H	1.258681278	3.762796480	-2.825039650
H	4.340525327	-1.298978400	-2.844345869
H	-1.684739628	6.133433160	-0.737605641
H	5.280284413	-4.955005702	-0.750054983
H	-0.380614670	-0.349010298	2.306054688
H	2.338742896	0.407698850	3.478569900
H	2.928183650	-2.482653785	2.378798500
H	-0.920433477	2.580356134	2.118722553
H	4.134699462	-4.069247399	1.262895223
H	5.372570185	-3.553542638	-2.810181843
H	-1.581347918	4.547612622	1.164516357
H	-0.258209226	5.723612836	-2.741003794

==============================

2

==============================

H	0.848536480	0.790385200	3.918172439
C	0.907389557	0.472025786	2.867498523
C	2.352635373	0.293556280	2.418277476
H	2.815736715	-0.579522054	2.900161301
N	0.274257298	1.436056410	1.978065616
C	-0.667071766	2.219000980	2.388112422
C	-1.416578258	3.117251167	1.570849865
C	-1.301319727	3.141160889	0.145659284
C	-2.160431518	4.011583986	-0.561166848
C	-3.064766134	4.814723899	0.096521247
C	-3.174270559	4.795995339	1.496715850
C	-2.360330378	3.949443449	2.209862203
O	-0.485742724	2.388119033	-0.530849444
Cr	1.051037705	1.465552815	0.115006133
O	1.644060008	0.957749169	-1.632621840
C	2.412494736	-0.032052593	-1.984308947
C	3.131277055	-0.864195647	-1.070759500
C	3.922676824	-1.922909577	-1.564502771
C	4.009932652	-2.193974359	-2.909165487
C	3.293958988	-1.384677717	-3.805773421
C	2.521571385	-0.334700166	-3.360742971
H	-2.070295250	4.021778287	-1.644125299
C	3.074934394	-0.689498346	0.346725890
N	2.338995658	0.165655538	0.970197551
H	1.963174351	0.296094485	-4.047805995
H	-3.892234761	5.434267294	2.003665596
H	4.617939951	-3.017740769	-3.271773009
H	0.378182503	-0.488280350	2.768887786
H	2.931711482	1.191584462	2.674049783
H	3.709289635	-1.363764204	0.941690774
H	-0.940910922	2.190798901	3.453549071
H	4.464392995	-2.538443460	-0.845954712
H	3.347882282	-1.588225849	-4.873586961
H	-2.432698740	3.906091462	3.296769292
H	-3.706400210	5.476216281	-0.482365826
O	-0.365768964	-0.498186715	-0.320114025
C	-0.689545754	-0.483214500	-1.447373868
O	-1.029034764	-0.510581328	-2.553143149
Cl	2.358448936	3.236900435	0.539937437

==============================

2-TS

==============================

C	1.379351110	0.377694359	-0.099400548
N	0.667030910	-0.034192422	1.894786589
H	1.167559131	0.097963073	-4.489820779
C	1.747979396	-0.102315460	-3.577752818
C	3.118336029	-0.685859101	-3.903103531
H	3.774588070	0.067996034	-4.362765504
N	1.054161189	-1.032281601	-2.695281849
C	-0.233591671	-1.119468805	-2.694433668
C	-1.016193558	-1.919698328	-1.806111522
C	-0.441793310	-2.673991134	-0.731886193
C	-1.333043907	-3.396590931	0.099122982
C	-2.694670862	-3.357560011	-0.101297222
C	-3.257860732	-2.600836762	-1.143294641
C	-2.417271154	-1.900245896	-1.975684729
O	0.821480179	-2.694935084	-0.449452873
Cr	2.297373322	-2.158082274	-1.546140316
O	3.640790526	-2.814209344	-0.367676271
C	4.860816502	-2.398902125	-0.230265120
C	5.532345260	-1.529218645	-1.148508623
C	6.869027110	-1.149440127	-0.902907069
C	7.551449056	-1.579489056	0.210125580
C	6.892156670	-2.429241255	1.114487709
C	5.592710043	-2.830884873	0.901994850
H	-0.889717319	-3.979102929	0.903754523
C	4.909506295	-1.025682775	-2.331847398
N	3.676036773	-1.200745893	-2.664287174
H	5.079044951	-3.494909100	1.593845960
H	-4.333949002	-2.574481700	-1.289619967
H	8.578492665	-1.272468017	0.386361855
H	1.874951382	0.852248806	-3.042824907
H	3.003975949	-1.528664277	-4.598988858
H	5.551157088	-0.434010824	-3.002953219
H	-0.797310224	-0.514383892	-3.420694137
H	7.356735298	-0.492506181	-1.623990985
H	7.418222675	-2.781604795	2.000175991
H	-2.827183479	-1.306116967	-2.792930965
H	-3.345692190	-3.925068931	0.561616127
O	2.377372963	-0.264351636	-0.154613902
O	0.533463503	1.107771649	-0.464172713
Cl	2.205754250	-3.891303965	-2.982261420
C	0.491888684	2.991938587	5.370364571
C	0.491048894	2.478263265	4.080699293
C	-0.386757691	1.451445551	3.722472410
C	-0.350617677	0.942919521	2.324390039
C	-1.272775516	0.957084120	4.677986629
C	-1.276721596	1.473630336	5.970119197
C	-0.393819128	2.488588584	6.319266606
C	-0.734938303	-0.429145952	1.945591148

H	1.095703580	-0.586762534	3.895507689
C	1.563665064	-0.622346173	2.895800745
C	1.944182667	-2.051850357	2.558347505
H	1.028812709	-2.646791026	2.421868013
C	2.808293412	-2.653864158	3.654169236
H	2.295781595	-2.652469209	4.625706685
H	3.075182594	-3.690162780	3.420977379
H	3.743696553	-2.091275070	3.774984053
H	2.468790305	-2.093240721	1.597109678
H	2.460229317	0.014420199	2.945445773
H	-1.214699807	-0.617862685	0.984488777
H	-1.002956656	-1.132166158	2.737129675
H	-0.492881332	1.710567721	1.559971413
H	1.181166433	2.869244503	3.332880345
H	1.183925022	3.787501843	5.637562134
H	-0.396592376	2.890761094	7.329836401
H	-1.974728661	1.081797580	6.706494357
H	-1.972150586	0.168652726	4.405406670

==============================

3

==============================

H	1.613419290	1.774367986	4.979158086
C	1.796486269	1.404517079	3.959583665
C	3.100752673	1.959290567	3.388186025
H	3.975462309	1.550524435	3.915617339
N	0.721631557	1.771803828	3.053530008
C	-0.458006724	2.073311836	3.465711008
C	-1.578877104	2.388963341	2.626915791
C	-1.555811762	2.230390486	1.205736264
C	-2.733010932	2.571238583	0.499628482
C	-3.863420057	3.019110873	1.152168073
C	-3.889803249	3.154348315	2.547423369
C	-2.754052352	2.836918395	3.259690866
O	-0.540029878	1.762004534	0.539104527
Cr	1.310018823	1.806559144	1.117645008
O	2.037585711	1.587734169	-0.649446464
C	3.183005744	1.068151580	-0.948969063
C	4.256148900	0.852013621	-0.020772507
C	5.467283576	0.287879123	-0.471617726
C	5.656870611	-0.087170252	-1.782127927
C	4.607509306	0.116999978	-2.692867922
C	3.414043184	0.677150065	-2.293709415
H	-2.701741023	2.478013607	-0.584097493
C	4.172906171	1.195294428	1.368687682
N	3.125750269	1.633208405	1.973380272
H	2.602080607	0.850600987	-2.996927892
N	-0.251775740	-1.794060344	0.154094342
C	-1.136716542	-2.935568235	0.464841073
C	-0.374092777	-4.060532378	1.138196160
C	-1.295866818	-5.232880522	1.437466113
C	-0.530541690	-0.924437877	-1.053652025
C	0.597604904	-1.860851705	-1.052547578
C	-1.816806118	-1.132679303	-1.760005384
C	-2.956163744	-0.555148276	-1.193718833
C	-4.194053096	-0.718048670	-1.799187738
C	-4.299884912	-1.452613518	-2.977991924
C	-3.167203919	-2.024142587	-3.547493762
C	-1.925368653	-1.868732405	-2.937765335
H	-4.783572992	3.506843013	3.054645809
H	6.596613822	-0.525451007	-2.106125401
H	1.849998804	0.305922651	3.993808398
H	3.104567964	3.054035238	3.483551458
H	5.094555617	1.055286513	1.954867195
H	-0.644742487	2.098308355	4.550129561
H	6.266879471	0.144908049	0.256275440
H	4.737916928	-0.167273417	-3.736071660
H	-2.746298083	2.935522149	4.345658715
H	-4.746183422	3.277750878	0.569377145
H	-0.292702016	0.117915256	-0.823057855
H	1.598656335	-1.459671760	-0.909489050
H	0.513020985	-2.807546644	-1.584936511
H	-1.035852154	-2.306605406	-3.388441209
H	-2.856655762	0.023880150	-0.273828210
H	-5.077323590	-0.267147594	-1.352488485
H	-3.249453049	-2.591980506	-4.471298791
H	-5.268949158	-1.576709175	-3.455812514
H	-1.598012760	-3.276713394	-0.470622099
H	-1.928852670	-2.546868465	1.114376699
H	0.073482883	-3.684344609	2.066002570
H	0.453155843	-4.382168765	0.488027362
H	-0.755922828	-6.049297054	1.926620665
H	-1.746486955	-5.634068615	0.521002366
H	-2.112907683	-4.930262745	2.103262520
O	1.262523576	-0.304783932	1.126107053
C	0.254749507	-0.959659839	1.428984634
O	-0.437832661	-1.132270345	2.400024597
Cl	1.453221096	4.086868608	1.167605858

==============================

3_{rot}

==============================

H	1.674515636	3.026768614	4.710944778
C	1.906217634	2.290296586	3.927304793
C	3.082241319	2.739461606	3.070376190
H	4.022856361	2.715732069	3.639674365
N	0.767399949	2.058819630	3.053332624
C	-0.446753590	2.254599101	3.429266705
C	-1.623023464	1.906180235	2.687164598
C	-1.585610125	1.211609684	1.435534943
C	-2.828035430	0.843763255	0.863520551
C	-4.024481116	1.154767449	1.470991913
C	-4.059956144	1.848099199	2.691072481
C	-2.868038869	2.207055019	3.277161829
O	-0.502824461	0.856645820	0.818101134
Cr	1.309217511	1.337162647	1.252420747
O	2.047762178	0.408182318	-0.271668723
C	3.280748949	0.398271402	-0.687844927
C	4.384075348	0.954108429	0.031212504
C	5.685126302	0.837465288	-0.493550977
C	5.936076808	0.208066558	-1.692502769
C	4.854032070	-0.325800467	-2.404889998
C	3.565922635	-0.234402357	-1.920183721
H	-2.788368267	0.301980671	-0.080233042
C	4.244930447	1.650726663	1.277211568
N	3.142349936	1.878605995	1.898013969
H	2.720583635	-0.631659091	-2.480567442
N	0.998433141	-2.430606630	1.098510733
C	-0.435846509	-2.266403409	1.436469765
C	-0.721753047	-2.610924545	2.884017871
C	-2.191489938	-2.367719554	3.193942571
C	1.391817961	-2.710818878	-0.324853649
C	1.486513187	-3.761130365	0.699287524
C	0.343423725	-2.669834157	-1.375136107
C	-0.144706296	-1.428271695	-1.796142229
C	-1.127518346	-1.375306401	-2.776136085
C	-1.616391127	-2.547406033	-3.346769910
C	-1.124155756	-3.780631877	-2.934771236
C	-0.148759759	-3.842487800	-1.945411178
H	-5.007322822	2.090583509	3.164180703
H	6.948127136	0.133260016	-2.079957772
H	2.155824700	1.334107192	4.410277443
H	2.906272879	3.766515826	2.718870603
H	5.178351738	2.038911923	1.713062300
H	-0.620357773	2.698383783	4.422084804
H	6.508107688	1.267410835	0.078152761
H	5.027641539	-0.817358869	-3.360730927
H	-2.867840777	2.734412557	4.231750028
H	-4.956643358	0.854436860	0.995291878
H	2.324988449	-2.192821389	-0.555910324
H	2.463863878	-3.997604041	1.110475954
H	0.723300816	-4.536909480	0.740609522
H	0.241392101	-4.807568536	-1.623605979
H	0.245989475	-0.516849472	-1.340778366

H	-1.511588110	-0.409120999	-3.095424318
H	-1.500394171	-4.697550443	-3.382711323
H	-2.383077718	-2.499121811	-4.116913516
H	-1.010629612	-2.912108847	0.760249755
H	-0.689278556	-1.224728260	1.209835448
H	-0.095727543	-1.986960865	3.533126171
H	-0.453199081	-3.660506391	3.080438845
H	-2.423506106	-2.605410671	4.236872788
H	-2.842489130	-2.982213216	2.558998046
H	-2.457897890	-1.316603746	3.019164505
O	3.181387093	-1.899799491	1.811056921
C	2.056876825	-1.472100791	1.854584406
O	1.473296515	-0.486471498	2.328431639
Cl	1.132901737	3.393741534	0.259815290

================================

3-TS$_{major}$

================================

O	-0.082220222	-0.072952311	-0.224550136
C	-0.441217210	-0.619771721	2.309652906
C	2.882402770	-0.312467567	1.785676643
C	5.196835262	-1.293996994	1.828799621
H	-1.417949098	2.002649272	-3.677155843
C	-0.937320916	1.243805987	-3.042158470
C	-0.333759340	0.113304310	-3.866615311
H	0.518753607	0.468550784	-4.464053487
N	-1.875123190	0.662950312	-2.094304941
C	-2.893975447	1.306620500	-1.656145401
C	-3.756195575	0.893343460	-0.582209798
C	-3.515243880	-0.271251648	0.217998874
C	-4.388004045	-0.483227589	1.316499341
C	-5.433529795	0.372828705	1.592581319
C	-5.673747961	1.502700024	0.797500143
C	-4.833559855	1.743580186	-0.267499658
O	-2.538862949	-1.104086330	0.043718446
Cr	-1.271045328	-1.155965538	-1.437035128
O	-0.372315216	-2.774989515	-0.844677872
C	0.838263509	-3.144922162	-1.119528304
C	1.667513701	-2.550366355	-2.124388322
C	2.990556673	-2.999710497	-2.298653735
C	3.529059893	-4.007145104	-1.527773636
C	2.713929170	-4.611293451	-0.558725152
C	1.412966663	-4.199379531	-0.362853849
H	-4.206168946	-1.370709474	1.920174238
C	1.207830529	-1.507488229	-2.997987306
N	0.063231435	-0.929191036	-2.936572469
H	0.773309534	-4.676192088	0.377788393
N	1.515406657	-0.458323471	1.319559457
C	3.773279932	-1.459517194	1.325057313
C	0.678465705	-1.496561573	1.919394857
C	-0.523457263	0.213613467	3.449375985
C	-1.651857109	1.060086881	3.540667346
C	-1.820611131	1.882044917	4.640947999
C	-0.868108802	1.873003927	5.656965784
C	0.251711948	1.037354059	5.587704834
C	0.425225685	0.209824634	4.497757281
H	-6.501013031	2.172167598	1.016267809
H	4.554551562	-4.334481459	-1.675720027
H	-0.132338300	1.720301716	-2.461845867
H	-1.095993185	-0.306512701	-4.537423249
H	1.912514955	-1.192281208	-3.782237308
H	-3.124007026	2.287742296	-2.101300591
H	3.595299918	-2.520825572	-3.069512922
H	3.112025948	-5.421593862	0.050647918
H	-4.989501182	2.622558555	-0.894263191
H	-6.084105942	0.161508642	2.440076803
H	-1.270770618	-0.583658193	1.594926732
H	0.362168715	-2.264176666	1.205668073
H	1.212471013	-1.953250995	2.759596614
H	1.286401231	-0.452780886	4.445900386
H	-2.385662051	1.045362098	2.732975379
H	-2.688827841	2.531283508	4.709926941
H	0.978589098	1.037801179	6.395392012
H	-0.998241591	2.520740812	6.521159427
H	2.882680601	-0.234624678	2.886839014
H	3.255754598	0.638263496	1.390291415
H	3.745194212	-1.484471593	0.226582112
H	3.354691088	-2.420844775	1.661895260
H	5.842426820	-2.106237217	1.479686343
H	5.237830857	-1.285470900	2.926308758
H	5.631925997	-0.350265024	1.476742580
O	2.036412910	0.605724762	-0.637459616
C	1.160833444	0.065205646	0.021336980
Cl	-2.746810368	-2.188017482	-2.866590501

================================

3-TS$_{minor}$

================================

H	0.863772031	3.967829036	4.092394860
C	1.173319138	2.999237669	3.671981319
C	2.516263562	3.105632374	2.960398360
H	3.335311724	3.283053222	3.672778407
N	0.199412457	2.490968996	2.721580566
C	-1.035926380	2.844153279	2.730996429
C	-2.076501536	2.280812174	1.921224509
C	-1.877151831	1.152903638	1.059186064
C	-3.020661230	0.648990912	0.390557817
C	-4.262415932	1.225063237	0.540651572
C	-4.451401009	2.338279625	1.374642682
C	-3.363645994	2.840947482	2.050930559
O	-0.749193388	0.539605233	0.889409496
Cr	0.992082954	1.121416449	1.479188094
O	1.933418598	-0.281092358	0.508719608
C	3.214331780	-0.416769438	0.355324854
C	4.209380343	0.358473191	1.034022812
C	5.571348875	0.075759673	0.831658374
C	6.001053470	-0.925584414	-0.014938890
C	5.034257308	-1.672594933	-0.697854923
C	3.686295020	-1.426749291	-0.521125552
H	-2.867139062	-0.212228086	-0.256435079
C	3.898389091	1.451373201	1.915163363
N	2.724334676	1.884883424	2.195862655
H	2.935718279	-1.990341351	-1.074773693
N	0.426821863	-2.233401635	2.627167180
C	-1.013293192	-2.026322086	2.694628370
C	-1.575918826	-2.104864021	4.105410242
C	-3.068540432	-1.819040261	4.106851190
C	2.296423863	-3.519846008	1.982458126
C	0.944029287	-3.513856786	2.438873887
H	-5.433811286	2.788240799	1.487045645
H	7.060285142	-1.119821521	-0.156457598
H	1.252989605	2.269057998	4.490000062
H	2.482165554	3.939823071	2.244517835
H	4.765756660	1.969653672	2.352944727
H	-1.348220510	3.632277732	3.434178632
H	6.302249069	0.682223097	1.367595602
H	5.345000750	-2.455687726	-1.388169150
H	-3.483528684	3.697331921	2.715624511
H	-5.110991223	0.807032667	0.001123343
H	0.683942252	-4.288726213	3.174788933
H	-1.486079144	-2.782100535	2.046817738
H	-1.230902919	-1.051362369	2.249788384
H	-1.046651410	-1.367214635	4.723649101
H	-1.368880065	-3.093924353	4.544111925
H	-3.487496493	-1.834603513	5.118334012
H	-3.617638779	-2.557287119	3.507669558
H	-3.273129175	-0.831210796	3.672605226
O	2.525595892	-1.621877478	3.278601278
C	1.360578569	-1.247718436	3.055071337
O	0.883108914	-0.077631947	3.158982170
Cl	1.141175954	2.675472148	-0.227548872
H	2.683824757	-2.733802273	1.341433001
H	2.953981725	-4.356304590	2.208832014
C	0.808684641	-4.116590887	0.997195077

C	0.551009822	-3.247405789	-0.082176987
C	0.815150964	-5.508780513	0.807839190
C	0.286819584	-3.785859434	-1.329919827
C	0.556895004	-6.031776226	-0.446824330
C	0.290103255	-5.169251050	-1.510764266
H	0.573383686	-2.165516689	0.081673173
H	1.014238415	-6.164012531	1.655650717
H	0.079851744	-3.124207748	-2.166956238
H	0.556888273	-7.107345250	-0.601872080
H	0.081733390	-5.582525115	-2.495174140

===============================

3-TS'$_{major}$

===============================

H	1.575815914	3.132072636	4.916732210
C	1.695562204	2.348079482	4.154050124
C	2.897781531	2.625606789	3.258693854
H	3.844309175	2.526342454	3.810183857
N	0.521735800	2.240166157	3.307115822
C	-0.639687228	2.660298292	3.667142048
C	-1.856700730	2.477777861	2.937495708
C	-1.942416498	1.666757927	1.757852200
C	-3.225798297	1.516738315	1.173054715
C	-4.338874334	2.123364373	1.708553470
C	-4.251083005	2.918126461	2.863869573
C	-3.021219543	3.077672873	3.458518938
O	-0.945143950	1.046970584	1.218630354
Cr	0.922171833	1.331091303	1.563838726
O	1.545435515	0.330299759	0.005201546
C	2.745321582	0.087294578	-0.367198721
C	3.927286115	0.446668004	0.374432471
C	5.187370954	0.084485462	-0.114942972
C	5.349418784	-0.583084666	-1.317635933
C	4.206530507	-0.893823057	-2.071824876
C	2.946855115	-0.594020840	-1.604123812
H	-3.284429443	0.906636042	0.274317804
C	3.890979438	1.236212197	1.580845951
N	2.837417251	1.715343509	2.126966855
H	2.052028130	-0.821070015	-2.180117838
N	0.963486370	-2.361647836	1.624821580
C	-0.460510084	-2.239333801	1.339444434
C	-1.340921798	-2.560541585	2.539037034
C	-2.810177981	-2.357242689	2.209968664
C	2.796811915	-3.349307734	0.531641867
C	1.666770328	-3.604125143	1.455635018
H	-5.135733975	3.393169497	3.278664363
H	6.343015717	-0.824485674	-1.687158643
H	1.836942241	1.381558922	4.658283661
H	2.822742748	3.646546397	2.857332674
H	4.870595725	1.470428826	2.025280865
H	-0.726107512	3.195131995	4.625815266
H	6.064186999	0.361789161	0.470801741
H	4.315608784	-1.382544228	-3.040067685
H	-2.925568038	3.682872411	4.360809088
H	-5.304056820	1.984881985	1.223980910
H	1.993962516	-4.048571105	2.406333385
H	0.991860517	-4.328278675	0.972885680
H	-0.696009971	-2.910722943	0.499745235
H	-0.660685413	-1.216457929	0.999823714
H	-1.040644337	-1.895519925	3.359657813
H	-1.153063547	-3.593506309	2.874948357
H	-3.454274807	-2.581358270	3.066910624
H	-3.128487047	-2.999161354	1.377526363
H	-2.996437615	-1.315422536	1.915615259
O	2.863233001	-1.586757946	2.575218415
C	1.672471745	-1.364557254	2.325814268
O	0.977279314	-0.342245603	2.653156346
Cl	0.944737947	3.372270771	0.457718569
H	2.506555231	-2.945074432	-0.442581817
C	4.157604258	-3.604039530	0.736235539
C	4.695592868	-3.911502146	2.014764701
C	5.020616928	-3.607132269	-0.389419202
C	6.021577428	-4.257008949	2.137604284
C	6.349427366	-3.968292665	-0.252974474
C	6.846901548	-4.294621813	1.005147910
H	4.063787598	-3.790180613	2.889248278
H	4.608764707	-3.360178960	-1.364189303
H	6.438123754	-4.477639839	3.116736299
H	7.002446710	-3.985773672	-1.121539100
H	7.895095575	-4.564208409	1.115363618

===============================

4$_{major}$

===============================

H	3.405837559	3.403701499	2.945838869
C	3.302003358	2.663119035	2.139470213
C	4.659696711	2.121393947	1.702026763
H	5.068727793	1.435582571	2.458231701
N	2.638311944	3.236218729	0.973882517
C	1.875110328	4.270152034	1.068163879
C	1.148537627	4.880226716	-0.005371673
C	1.116735074	4.342403619	-1.331080534
C	0.315431675	5.015082193	-2.282352515
C	-0.393456492	6.149949518	-1.954460576
C	-0.354327798	6.681931322	-0.656169249
C	0.407364374	6.041710565	0.293886615
O	1.743874023	3.265149470	-1.699477863
Cr	3.210706003	2.412939076	-0.793666021
O	3.690652841	1.307446078	-2.265481041
C	4.343195088	0.186521141	-2.249393080
C	4.973694056	-0.369997534	-1.091345211
C	5.614020313	-1.623762039	-1.175797246
C	5.659380852	-2.337145717	-2.351002304
C	5.067228103	-1.777997747	-3.495567224
C	4.440528640	-0.552987376	-3.451737934
H	0.299577422	4.604443582	-3.289429464
C	5.037087623	0.319092572	0.160602810
N	4.491849438	1.456322087	0.420639034
H	3.967895705	-0.123286899	-4.331943851
N	0.145964008	-0.521935085	0.749214259
C	-0.360639695	-1.430990609	1.756109814
C	-0.235644794	-2.890883558	1.343616433
C	-0.797504618	-3.825093081	2.401483386
C	0.492979971	0.557259312	-1.272537446
C	-0.610783069	-0.029029922	-0.377214386
C	0.836356271	-0.294362102	-2.457793183
C	1.128404069	-1.652426449	-2.311130797
C	1.378779196	-2.438200815	-3.427613750
C	1.334870277	-1.873934973	-4.699607370
C	1.063127157	-0.518792029	-4.849580495
C	0.822916249	0.270865449	-3.729577547
H	-0.912678034	7.580040388	-0.408061032
H	6.152461616	-3.304387767	-2.396457584
H	2.697076093	1.823161346	2.504833240
H	5.362436322	2.953781907	1.556528030
H	5.617974842	-0.173259881	0.954651752
H	1.750083745	4.738165667	2.057108665
H	6.082327291	-2.021487469	-0.274811372
H	5.099022009	-2.322340375	-4.438670001
H	0.451985424	6.430419345	1.311721457
H	-0.989429855	6.643866881	-2.719891000
H	0.267168213	1.576828157	-1.596354489
H	-1.152017240	-0.835060600	-0.889754731
H	-1.341956444	0.729899766	-0.060826732
H	0.640352561	1.339868322	-3.832504765
H	1.164130846	-2.098634506	-1.316764827
H	1.611717344	-3.493699873	-3.306257398
H	1.045534137	-0.071375407	-5.840799588
H	1.525139054	-2.491282565	-5.575071106
H	-1.410045796	-1.171014397	1.960235872
H	0.207523815	-1.249338853	2.677253151
H	0.829108776	-3.103455484	1.167138111
H	-0.751119678	-3.046187175	0.383836869
H	-0.690625589	-4.874776609	2.111253905

H	-1.864707638	-3.635766431	2.573152967
H	-0.280996490	-3.691726773	3.360082897
O	1.635465168	0.675394909	-0.370265909
C	1.428388918	-0.079140821	0.757519693
O	2.277444844	-0.280632597	1.593499591
Cl	4.691143769	4.087615386	-1.149662084

==============================

4$_{minor}$

==============================

H	0.813823767	3.861952152	4.173242474
C	1.172883895	2.924221507	3.723639368
C	2.461083475	3.140612049	2.937001953
H	3.303389940	3.377898823	3.603190713
N	0.189850278	2.351588563	2.818727400
C	-1.067864809	2.613581289	2.896641778
C	-2.095530031	2.015411054	2.097480306
C	-1.851696283	0.922195290	1.203846563
C	-2.963709764	0.412899550	0.490345866
C	-4.227817356	0.937302515	0.651835611
C	-4.467568559	1.996123844	1.541995705
C	-3.405989410	2.513434318	2.248275562
O	-0.701578393	0.344742752	1.047112706
Cr	1.015022051	1.078074068	1.490859835
O	1.983739184	-0.260268720	0.493184611
C	3.267856602	-0.360274745	0.328004079
C	4.239655715	0.482178384	0.957374877
C	5.610865100	0.259416746	0.723036817
C	6.059128415	-0.749929178	-0.098560875
C	5.109842602	-1.574157207	-0.719180665
C	3.758918858	-1.387632167	-0.514606975
H	-2.769586336	-0.405313241	-0.200313671
C	3.902010238	1.573114542	1.825561487
N	2.715001225	1.939937214	2.155203212
H	3.021035664	-2.019698763	-1.006323493
N	0.493287235	-2.424661667	2.748351227
C	-0.961589875	-2.269671929	2.709848461
C	-1.529521521	-1.523192991	3.905121699
C	-3.046144914	-1.465669292	3.824355438
C	2.633926584	-3.238978153	2.429667770
C	1.150639508	-3.667061462	2.365179609
H	-5.468100844	2.400872558	1.665417759
H	7.121638516	-0.899411051	-0.267950990
H	1.356339086	2.198999845	4.529880776
H	2.320687410	3.974568119	2.233946064
H	4.754011209	2.146411142	2.223041514
H	-1.402619203	3.357550582	3.636314609
H	6.326636829	0.919262667	1.214458688
H	5.440592159	-2.373396222	-1.380542166
H	-3.564259683	3.338148600	2.944060423
H	-5.053461380	0.520025306	0.077671907
H	0.927727337	-4.448449038	3.109915828
H	-1.371301577	-3.289423588	2.672717131
H	-1.246649846	-1.766822318	1.775742202
H	-1.114007123	-0.508596701	3.924761313
H	-1.203883360	-2.018264333	4.831899287
H	-3.470434182	-0.918341001	4.672208171
H	-3.489253128	-2.470262484	3.820630181
H	-3.368288984	-0.955134591	2.906457388
O	2.633141404	-1.894684264	2.916274055
C	1.376105751	-1.434753693	2.956837698
O	1.134680337	-0.248166484	3.186368911
Cl	0.943778709	2.642952010	-0.172005963
H	3.099934845	-3.234405186	1.435593266
H	3.231265381	-3.853562936	3.108767958
C	0.728813173	-4.153320501	1.002998311
C	0.618661460	-3.250727544	-0.058405368
C	0.473502887	-5.504684463	0.789888645
C	0.269701509	-3.711293683	-1.321648570
C	0.129911016	-5.963551389	-0.478448997
C	0.028971797	-5.066714292	-1.535143112
H	0.804577642	-2.185416833	0.103094601
H	0.548101226	-6.204583375	1.622627812
H	0.183904717	-3.003639058	-2.143557613
H	-0.063637835	-7.021857754	-0.638311494
H	-0.242935865	-5.422053024	-2.526563282

Table S2. Vibrational frequencies of all structures investigated

==============================

1

==============================

22.78	49.05	58.34	73.12	99.95	104.00
116.50	146.81	177.55	208.38	221.02	235.13
252.68	298.45	311.28	329.89	345.98	353.13
354.03	374.66	393.00	414.55	456.33	465.17
472.77	488.37	532.35	552.88	554.55	581.21
599.69	626.80	629.95	643.00	651.02	749.63
751.15	766.08	767.12	815.33	818.79	861.87
869.92	871.01	905.86	909.80	939.17	940.44
968.44	985.57	989.26	989.76	990.55	1053.25
1055.44	1075.24	1105.59	1111.10	1145.38	1147.68
1156.56	1157.35	1219.64	1240.84	1243.49	1252.85
1262.49	1270.47	1336.71	1358.90	1373.07	1377.22
1399.71	1406.32	1424.75	1430.06	1449.52	1457.41
1497.39	1505.01	1507.60	1508.52	1593.37	1595.24
1681.08	1682.04	1695.01	1708.78	3009.07	3019.67
3047.12	3047.68	3068.04	3085.53	3156.14	3156.23
3173.31	3173.53	3200.65	3200.70	3205.33	3205.63

==============================

2

==============================

17.18	37.10	49.49	56.51	60.49	78.10
91.72	104.31	107.50	120.45	131.08	144.53
174.13	189.81	215.91	222.93	249.55	263.07
300.12	317.18	335.38	346.93	354.29	364.84
377.10	395.58	407.90	459.73	465.01	469.44
490.74	537.96	552.44	553.94	585.00	600.02
626.27	629.51	644.37	653.68	656.70	662.82
749.53	750.29	764.22	765.25	817.43	820.04
866.81	867.33	868.98	905.00	909.19	937.01
938.23	965.24	984.62	987.01	987.57	989.47
1057.38	1058.71	1076.09	1107.75	1113.80	1145.94
1148.74	1157.27	1157.90	1217.47	1240.90	1244.08
1251.55	1264.12	1271.37	1334.97	1355.90	1372.18
1375.34	1396.93	1399.95	1405.77	1425.46	1429.26
1448.10	1454.03	1502.38	1508.07	1509.07	1509.33
1597.19	1598.39	1683.47	1685.12	1705.91	1719.61
2502.58	3008.46	3021.30	3046.80	3047.37	3069.61
3088.90	3155.01	3155.87	3172.74	3173.02	3198.66

==============================

2-TS

==============================

-114.84	15.69	19.27	28.14	28.81	38.14
46.07	54.65	61.73	73.23	76.31	83.86
85.04	86.75	98.17	113.41	122.59	126.14
140.01	153.09	160.45	170.44	179.04	191.75
217.20	218.57	225.67	245.73	253.36	262.54
266.18	295.94	309.32	320.55	337.58	343.33
345.97	354.48	369.47	373.89	387.08	398.59
403.45	410.50	453.59	458.84	465.61	468.14
477.86	491.14	527.53	538.75	552.82	553.74
565.97	584.60	599.72	620.71	624.74	625.36
629.17	643.47	653.60	700.79	707.96	733.55
747.08	750.07	751.03	762.81	764.46	792.46
815.86	817.20	838.82	855.67	863.84	865.17
865.71	882.19	905.06	909.62	913.83	927.15
932.73	934.90	938.04	964.62	964.67	984.33
984.67	985.16	986.71	989.45	995.40	1012.32

1044.69 1055.80 1057.08 1057.22 1075.85 1076.31
1099.22 1105.85 1112.72 1113.61 1124.29 1138.76
1144.78 1146.08 1147.92 1155.96 1156.59 1163.72
1185.13 1194.16 1218.26 1233.67 1237.04 1239.60
1251.39 1263.47 1270.44 1276.09 1308.54 1318.09
1320.80 1330.53 1335.89 1345.38 1354.49 1376.08
1377.55 1380.26 1396.28 1398.68 1404.05 1404.76
1412.47 1424.86 1428.99 1448.15 1452.00 1457.73
1465.74 1476.47 1478.44 1483.79 1491.93 1507.16
1507.23 1509.13 1514.58 1540.90 1593.30 1594.77
1657.35 1681.54 1682.12 1683.74 1706.27 1719.84
2322.89 2984.63 3006.20 3017.70 3018.81 3038.02
3042.58 3046.46 3052.67 3066.67 3085.37 3092.48
3097.23 3120.64 3122.02 3128.83 3151.81 3154.87
3158.59 3167.69 3167.98 3170.83 3178.40 3186.81
3190.29 3192.00 3193.08 3196.82 3200.88 3201.58
3199.69 3203.99 3204.67

================================
3
================================
4.95 19.67 27.80 35.19 39.62 55.97
59.28 62.93 65.71 77.25 86.92 89.45
100.00 108.97 117.50 126.41 134.87 150.07
161.44 173.02 190.23 200.58 214.01 225.23
237.09 245.01 254.49 262.11 277.17 295.04
301.55 319.90 336.70 338.72 344.04 355.17
358.46 378.69 380.44 397.14 408.04 413.96
414.52 453.53 459.95 467.16 469.75 489.67
497.53 538.00 543.13 554.27 556.16 580.54
585.13 595.89 619.27 621.34 623.49 623.82
643.24 653.10 709.96 721.10 734.86 739.81
749.29 752.11 760.37 761.67 783.42 794.96
810.28 813.66 834.07 859.29 860.78 863.68
867.20 867.80 891.81 902.48 908.15 923.58
932.05 932.63 934.91 958.04 975.79 979.16
979.79 980.42 985.94 995.06 1001.99 1007.06
1036.53 1052.69 1056.60 1060.03 1069.56 1075.66
1100.04 1106.07 1114.38 1119.17 1139.24 1142.84
1144.17 1146.48 1154.79 1155.54 1164.47 1165.77
1185.34 1208.83 1214.31 1231.24 1236.17 1244.74
1249.75 1264.15 1270.34 1284.86 1309.85 1317.95
1325.75 1333.91 1336.75 1341.60 1350.10 1372.02
1377.91 1378.74 1395.52 1397.76 1399.85 1404.63
1412.33 1417.73 1423.72 1446.66 1447.24 1453.30
1468.64 1470.42 1478.58 1486.46 1490.23 1504.38
1505.98 1510.07 1518.86 1540.40 1591.45 1599.08
1655.87 1678.16 1679.47 1681.02 1721.03 1730.21
1987.60 3011.04 3016.12 3027.13 3033.58 3037.61
3044.00 3058.89 3068.65 3085.30 3092.78 3115.01
3116.53 3123.29 3130.02 3133.06 3149.32 3150.83
3152.56 3162.27 3164.41 3166.00 3176.39 3186.19
3187.17 3187.58 3196.44 3199.03 3199.22 3230.97

================================
3_{rot}
================================
14.77 22.61 35.91 43.08 52.33 56.89
65.31 67.53 75.11 83.87 86.86 91.90
101.74 104.36 111.23 127.56 140.52 145.93
166.64 174.24 194.11 195.81 208.46 237.35
240.36 252.28 259.16 264.23 275.20 290.82
305.98 314.08 325.90 329.37 338.57 349.96
355.48 366.47 371.16 392.65 407.89 408.40
413.59 454.78 458.31 471.03 476.00 486.61
494.76 529.79 537.06 555.30 556.23 574.54
585.52 595.27 616.81 619.51 622.60 625.15
639.89 650.92 709.14 712.14 735.83 743.43
751.50 754.50 761.87 762.32 788.19 793.79
798.87 811.65 838.30 861.71 863.47 865.84
866.98 877.57 893.86 903.09 907.60 927.56
934.78 937.08 937.46 963.91 979.55 982.34

982.45 983.14 989.03 989.55 1002.20 1007.21
1035.07 1054.36 1057.16 1064.18 1068.65 1075.18
1104.26 1108.98 1112.83 1120.22 1130.89 1143.67
1144.65 1147.45 1156.58 1157.89 1164.60 1164.80
1192.47 1206.53 1221.13 1233.34 1234.84 1236.84
1255.19 1265.75 1272.92 1287.34 1302.23 1314.64
1328.30 1336.66 1336.81 1343.37 1359.11 1374.76
1376.51 1379.33 1398.70 1401.54 1403.51 1404.22
1405.72 1420.66 1427.05 1444.65 1450.63 1450.98
1468.34 1469.45 1476.89 1484.70 1485.40 1504.82
1507.25 1512.69 1517.29 1539.03 1595.94 1601.09
1654.11 1680.28 1681.00 1682.99 1719.46 1729.03
1992.57 3012.67 3015.80 3019.29 3026.12 3038.76
3041.85 3057.91 3069.00 3082.59 3092.09 3110.77
3129.37 3130.95 3137.18 3140.16 3152.52 3153.85
3163.15 3164.45 3164.75 3167.74 3176.17 3179.65
3180.65 3187.24 3196.33 3200.80 3201.35 3243.59

================================
$3\text{-}TS_{major}$
================================
-145.24 15.80 21.53 30.98 33.91 45.16
50.56 60.49 69.65 75.53 80.04 88.66
96.32 104.97 108.11 112.78 123.79 130.88
139.47 156.95 177.70 182.55 192.49 216.56
233.03 235.84 242.03 263.02 269.35 294.49
304.18 314.18 319.29 329.10 337.72 346.03
357.49 369.96 385.72 393.30 404.44 408.12
409.38 437.07 446.93 458.07 469.18 471.91
482.53 489.47 539.61 540.65 554.63 557.84
583.36 593.46 596.69 614.54 615.38 621.97
643.71 653.17 656.93 676.23 724.13 748.75
749.63 759.30 764.18 771.42 798.56 802.06
808.45 811.08 828.59 848.78 860.11 860.66
867.87 868.91 902.13 902.67 907.66 929.86
933.95 935.34 961.28 965.85 974.53 974.69
979.21 985.38 988.90 989.22 1004.52 1016.83
1040.79 1050.55 1057.73 1059.58 1064.16 1075.01
1102.14 1107.87 1113.84 1115.43 1128.51 1142.24
1145.70 1154.78 1155.40 1159.01 1170.80 1189.59
1209.70 1220.83 1229.98 1233.23 1255.06 1264.34
1272.35 1273.57 1280.96 1293.50 1305.70 1316.72
1331.38 1337.35 1344.96 1350.55 1359.39 1375.43
1377.06 1388.87 1395.84 1399.71 1400.03 1403.90
1417.28 1422.64 1431.78 1444.11 1447.19 1450.35
1458.47 1465.74 1477.47 1482.21 1496.58 1503.07
1506.54 1516.62 1523.81 1556.19 1592.64 1595.10
1629.37 1665.57 1675.76 1678.61 1727.88 1736.25
1820.64 2989.97 3009.27 3017.98 3018.35 3025.16
3029.24 3046.70 3066.73 3070.71 3073.66 3088.49
3105.02 3115.30 3120.99 3126.21 3141.22 3144.49
3148.70 3152.14 3160.68 3160.98 3177.69 3181.31
3182.41 3188.00 3190.58 3196.94 3201.14 3205.90

================================
$3\text{-}TS'_{major}$
================================
-142.11 8.62 14.65 36.36 38.41 48.03
51.25 64.68 71.81 79.66 83.29 93.61
97.73 103.93 116.34 129.55 136.76 143.27
149.45 160.87 169.71 180.99 192.68 213.34
228.82 242.38 252.48 257.70 267.54 288.76
293.10 310.99 318.04 322.61 328.53 347.98
355.56 365.47 376.13 381.88 395.24 403.27
406.84 434.75 451.58 460.61 466.57 470.07
481.63 487.61 535.66 550.66 553.78 555.38
578.87 582.96 606.53 614.37 616.23 619.14
637.24 647.34 661.11 673.09 735.11 739.46
748.63 758.30 759.34 761.18 768.76 787.34
796.58 823.80 827.88 848.11 855.80 861.84
862.28 869.26 884.49 897.77 905.00 911.42
926.42 934.48 934.98 959.56 964.48 971.15

974.24	977.36	980.81	984.32	1002.41	1004.07
1048.35	1050.89	1053.24	1062.77	1073.02	1082.95
1098.78	1107.67	1114.77	1122.89	1123.12	1135.01
1145.50	1152.10	1154.99	1167.81	1174.65	1189.05
1217.96	1221.40	1228.69	1234.20	1252.18	1255.09
1264.18	1271.43	1279.36	1297.18	1306.01	1315.92
1333.58	1337.23	1352.43	1355.71	1378.56	1379.47
1389.05	1394.82	1398.55	1401.25	1402.23	1407.34
1416.96	1425.34	1426.94	1441.87	1448.21	1459.10
1463.24	1469.17	1476.03	1485.30	1488.50	1507.67
1508.60	1514.53	1535.17	1554.63	1578.50	1592.18
1616.13	1655.52	1660.67	1680.58	1718.40	1721.31
1736.02	3003.27	3009.34	3013.27	3015.43	3017.87
3021.01	3038.87	3039.47	3066.72	3071.47	3077.15
3084.94	3100.29	3113.47	3120.30	3140.72	3150.78
3153.60	3156.45	3166.23	3179.55	3188.24	3190.24
3192.84	3193.85	3199.41	3199.56	3210.72	3221.50

==============================

3-TS_{minor}

==============================

-465.66	3.98	18.85	36.25	42.11	47.59
59.07	59.97	68.48	80.70	86.05	99.29
103.33	108.55	118.41	123.32	133.28	144.28
151.74	171.86	192.22	195.30	210.06	213.38
235.63	254.59	256.28	263.60	270.95	294.58
296.57	310.83	313.90	320.60	326.40	338.81
354.23	356.38	367.07	374.58	395.14	406.01
407.71	417.18	438.41	457.31	468.84	475.93
486.59	518.95	536.23	555.74	558.16	582.35
584.71	591.69	612.27	615.66	621.06	639.79
640.29	650.09	677.40	681.94	735.79	744.51
747.31	748.47	757.70	759.94	773.74	800.86
804.85	818.51	838.56	852.75	859.47	859.59
861.03	865.38	868.00	902.26	907.53	913.18
929.85	933.09	953.99	962.30	968.98	971.45
977.75	979.77	986.45	997.22	1003.45	1022.53
1028.86	1048.15	1055.16	1059.87	1062.02	1063.67
1074.60	1107.73	1107.89	1113.94	1118.25	1128.54
1142.74	1146.40	1154.65	1155.20	1157.20	1169.24
1192.43	1220.91	1226.16	1229.70	1232.49	1239.63
1255.36	1264.86	1266.10	1272.04	1298.60	1311.27
1326.21	1336.92	1337.87	1358.82	1375.38	1377.49
1381.08	1394.18	1400.73	1402.63	1404.51	1412.53
1418.20	1425.53	1428.52	1443.21	1448.63	1459.49
1469.88	1478.66	1479.04	1486.29	1496.80	1503.22
1506.88	1512.57	1517.73	1523.60	1594.93	1597.57
1636.74	1654.74	1675.72	1681.93	1713.04	1726.91
1742.96	2997.32	3001.97	3012.61	3013.96	3014.53
3032.02	3035.26	3036.52	3068.56	3069.39	3082.74
3095.15	3114.17	3121.26	3124.08	3148.86	3149.78
3151.75	3158.18	3163.22	3164.91	3174.36	3181.54
3186.42	3197.15	3198.88	3201.28	3202.74	3272.33

==============================

4_{major}

==============================

6.15	15.45	24.13	37.17	40.37	46.80
47.20	56.47	70.07	78.36	84.47	90.10
95.82	100.92	108.32	116.29	124.45	139.22
146.14	164.75	188.76	195.70	224.41	226.28
227.55	241.97	248.55	267.93	273.40	300.88
302.27	317.61	322.79	331.61	336.23	344.08
356.30	368.27	372.27	393.55	394.01	401.30
407.18	461.06	467.29	470.08	490.28	512.31
540.29	553.21	554.78	582.96	585.33	598.24
600.88	621.91	624.46	631.54	642.79	654.97
685.65	705.29	738.90	745.44	749.06	753.67
760.43	763.67	767.11	787.88	799.50	814.67
816.18	835.25	855.12	859.46	861.80	864.69
868.62	904.18	905.38	909.69	925.78	932.54
934.30	964.10	966.62	974.81	981.65	983.14
988.47	993.13	997.80	1006.70	1011.51	1056.10
1056.87	1059.21	1062.56	1067.13	1075.12	1076.78
1106.71	1110.77	1113.65	1127.63	1144.40	1147.08
1152.15	1154.98	1156.40	1164.14	1189.42	1190.47
1227.50	1235.28	1237.23	1240.71	1243.76	1255.21
1263.98	1272.80	1287.72	1306.43	1312.08	1319.37
1327.87	1339.28	1360.44	1365.42	1368.95	1374.89
1376.08	1398.32	1399.03	1401.29	1404.01	1404.87
1421.53	1427.05	1442.57	1444.54	1452.47	1467.06
1470.16	1478.47	1486.05	1492.53	1505.02	1506.67
1508.51	1510.77	1513.92	1531.87	1594.30	1598.00
1654.78	1678.13	1681.55	1683.25	1710.96	1722.47
1895.44	3011.79	3014.16	3018.31	3023.88	3024.82
3030.35	3039.02	3049.40	3063.58	3076.83	3078.89
3096.86	3096.95	3110.53	3122.35	3131.34	3141.33
3150.95	3153.19	3154.70	3161.13	3165.01	3168.82
3171.17	3185.91	3190.56	3192.73	3198.11	3202.23

==============================

4_{minor}

==============================

8.21	23.89	34.30	41.88	53.01	55.83
61.86	63.46	72.18	87.71	93.65	99.32
105.42	117.35	121.79	130.19	152.43	156.46
170.65	177.79	193.33	205.13	212.57	230.18
245.97	252.63	256.69	271.47	283.57	291.04
296.46	305.64	315.06	333.67	340.37	346.58
355.11	365.84	372.54	391.16	407.25	410.27
416.24	458.53	466.67	474.99	490.20	507.71
537.43	555.29	556.62	571.82	584.83	594.98
609.03	619.99	621.41	623.96	640.37	649.55
710.48	717.47	725.27	741.45	750.76	751.60
761.10	762.80	772.05	781.99	790.15	804.65
808.55	861.10	863.77	864.92	866.34	869.24
878.01	903.36	908.31	921.50	932.16	933.27
935.75	962.08	980.45	980.94	982.21	982.84
984.14	988.15	1006.95	1015.50	1056.49	1057.31
1060.30	1063.28	1069.84	1074.75	1086.29	1106.35
1112.76	1114.39	1126.67	1139.43	1144.81	1148.09
1155.88	1157.89	1164.04	1180.59	1196.14	1218.29
1219.99	1232.04	1232.56	1235.16	1243.58	1253.12
1265.10	1269.65	1271.79	1289.09	1317.28	1319.04
1335.11	1341.52	1356.27	1361.44	1374.79	1376.02
1379.54	1392.19	1397.64	1402.30	1403.38	1405.14
1419.90	1425.60	1444.75	1447.00	1451.74	1472.26
1475.68	1480.48	1488.43	1491.57	1504.75	1506.53
1510.59	1515.60	1520.55	1533.69	1594.55	1596.49
1659.96	1678.01	1679.79	1681.73	1718.15	1725.61
1787.12	3003.49	3012.36	3014.93	3020.32	3026.03
3035.16	3038.02	3038.92	3048.46	3068.65	3081.91
3086.55	3096.40	3112.33	3123.79	3125.13	3134.29
3151.54	3151.77	3152.95	3165.35	3165.98	3170.90
3180.74	3183.96	3187.29	3191.85	3200.42	3200.67

S9. Author contributions audit. DA, AWM, STN conceived the idea for the project. DA completed all the computations under the supervision of M-HB. AWM carried out the experiments under the supervision of STN and wrote an initial draft of the manuscript. DA and STN revised the manuscript with input from co-authors.

S10. References

S1. Jaguar 7.0, Schrödinger, Inc., Portland, Oregon, 2003.
S2. Y. Zhao and D. Truhlar, *Theor. Chem. Acc.*, 2008, **120**, 215-241.
S3. P. J. Hay and W. R. Wadt, *J. Chem. Phys.*, 1985, **82**, 270-283.
S4. W. R. Wadt and P. J. Hay, *J. Chem. Phys.*, 1985, **82**, 284-298.
S5. T. H. Dunning, Jr., *J. Chem. Phys.*, 1989, **90**, 1007-1023.
S6. B. Marten, K. Kim, C. Cortis, R. A. Friesner, R. B. Murphy, M. N. Ringnalda, D. Sitkoff and B. Honig, *J. Phys. Chem.*, 1996, **100**, 11775-11788.
S7. M. Friedrichs, R. H. Zhou, S. R. Edinger and R. A. Friesner, *J. Phys. Chem. B*, 1999, **103**, 3057-3061.
S8. S. R. Edinger, C. Cortis, P. S. Shenkin and R. A. Friesner, *J. Phys. Chem. B*, 1997, **101**, 1190-1197.
S9. A. A. Rashin and B. Honig, *J. Phys. Chem.*, 1985, **89**, 5588-5593.
S10. T. A. Halgren and W. N. Lipscomb, *Chem. Phys. Lett.*, 1977, **49**, 225-232.
S11. C. Y. Peng and H. B. Schlegel, *Isr. J. Chem.*, 1993, **33**, 449-454.
S12. P. Brandt, P.-O. Norrby, A. M. Daly and D. G. Gilheany, *Chem.–Eur. J.*, 2002, **8**, 4299-4307.
S13. R. L. Paddock and S. T. Nguyen, *J. Am. Chem. Soc.*, 2001, **123**, 11498-11499.
S14. L. E. Martinez, J. L. Leighton, D. H. Carsten and E. N. Jacobsen, *J. Am. Chem. Soc.*, 1995, **117**, 5897-5898.
S15. I. Okada, K. Ichimura and R. Sudo, *Bull. Chem. Soc. Jpn.*, 1970, **43**, 1185-1189.
S16. F. H. Vonderheiden and J. W. Eldridge, *J. Chem. Eng. Data*, 1963, **8**, 20-21.
S17. R. G. Konsler, J. Karl and E. N. Jacobsen, *J. Am. Chem. Soc.*, 1998, **120**, 10780-10781.
